# The biofilm matrix scaffold of *Pseudomonas aeruginosa* contains G-quadruplex extracellular DNA structures

**DOI:** 10.1038/s41522-021-00197-5

**Published:** 2021-03-19

**Authors:** Thomas Seviour, Fernaldo Richtia Winnerdy, Lan Li Wong, Xiangyan Shi, Sudarsan Mugunthan, Yong Hwee Foo, Remi Castaing, Sunil S. Adav, Sujatha Subramoni, Gurjeet Singh Kohli, Heather M. Shewan, Jason R. Stokes, Scott A. Rice, Anh Tuân Phan, Staffan Kjelleberg

**Affiliations:** 1 Singapore Centre for Environmental Life Sciences Engineering, Nanyang Technological University, Singapore, Singapore; 2 WATEC Aarhus University Centre for Water Technology, Aarhus, Denmark; 3 School of Physical and Mathematical Sciences, Nanyang Technological University, Singapore, Singapore; 4 Materials and Chemical Characterisation Facility (MC2), University of Bath, Bath, UK; 5 Singapore Phenome Centre, Nanyang Technological University, Singapore, Singapore; 6 School of Chemical Engineering, University of Queensland, Brisbane, QLD Australia; 7 The iThree Institute, University of Technology Sydney, Sydney, NSW Australia; 8 School of Biological Sciences, Nanyang Technological University, Singapore, Singapore; 9 School of Biological, Earth and Environmental Sciences, University of New South Wales, Sydney, NSW Australia

**Keywords:** Biofilms, Pathogens

## Abstract

Extracellular DNA, or eDNA, is recognised as a critical biofilm component; however, it is not understood how it forms networked matrix structures. Here, we isolate eDNA from static-culture *Pseudomonas aeruginosa* biofilms using ionic liquids to preserve its biophysical signatures of fluid viscoelasticity and the temperature dependency of DNA transitions. We describe a loss of eDNA network structure as resulting from a change in nucleic acid conformation, and propose that its ability to form viscoelastic structures is key to its role in building biofilm matrices. Solid-state analysis of isolated eDNA, as a proxy for eDNA structure in biofilms, reveals non-canonical Hoogsteen base pairs, triads or tetrads involving thymine or uracil, and guanine, suggesting that the eDNA forms G-quadruplex structures. These are less abundant in chromosomal DNA and disappear when eDNA undergoes conformation transition. We verify the occurrence of G-quadruplex structures in the extracellular matrix of intact static and flow-cell biofilms of *P. aeruginosa*, as displayed by the matrix to G-quadruplex-specific antibody binding, and validate the loss of G-quadruplex structures in vivo to occur coincident with the disappearance of eDNA fibres. Given their stability, understanding how extracellular G-quadruplex structures form will elucidate how *P. aeruginosa* eDNA builds viscoelastic networks, which are a foundational biofilm property.

## Introduction

Biofilms are key microbial ecosystems that, for example, contribute to bacterial pathogenicity^1^, disrupt the flow in water filtration systems^2^ and facilitate wastewater treatment bioprocesses^3^. They represent bacterial adaptation strategies allowing for increased antibiotic tolerance^4^, enhanced resource capture^5^ and the establishment of ecological microniches^6^. Such properties are unique to biofilms, in contrast to planktonic bacteria, and are not mediated directly by the microbial cells, but instead by the extracellular polymeric matrix the cells produce^7^. While cells themselves are stiff and rigid, the matrix provides the biofilm with a viscoelasticity that allows microorganisms to withstand mechanical and chemical stresses, and enhances surface colonisation by facilitating biofilm migration. Moreover, the viscoelastic property of the matrix is recognised as a specific virulence factor in chronic biofilm infections^8^.

Exopolymer functions in biofilms have been studied extensively, particularly for *Pseudomonas aeruginosa*, which contributes to one in five clinical infections^9^. No fewer than eight exopolymers have been identified as supporting key traits in *P. aeruginosa* biofilms, including three exopolysaccharides^10^, four proteins^11–13^ and extracellular DNA (eDNA)^14^. While progress has been made towards describing the structures and identities of extracellular polysaccharides and proteins by applying classical chemical and molecular approaches, important questions regarding eDNA remain, for example, how does it differ structurally from chromosomal DNA (cDNA) and what enables it to induce structure-dependent functions in the biofilm matrix? eDNA has been described as a key matrix biopolymer in clinical^15^ and environmental biofilms^16^, particularly in *P. aeruginosa* biofilms^13^, colocalises with the polysaccharide Pel^17^ and undergo degradation by DNA-specific endonucleases^18^. While there have been attempts to explain the properties of eDNA, they have focused on primary-structure differences with the cDNA and, as yet, no signature eDNA sequences have been identified^19^.

DNA can form multiple higher-order structures arising from differences in torsional stress as the two strands are twisted around the axis. cDNA consists of supercoiled duplex structures that are formed by the action of histone-like proteins and topoisomerases, and then relaxed by gyrases to allow replication and transcription to occur^20^. DNA supercoiling, however, does not reconcile with descriptions of eDNA as a primary biofilm structural agent, implying a networked structure. While eDNA colocalises with DNA-binding proteins^21^, it does not distinguish it from cDNA nor allude to its higher-order structure. Thus, there is increasing interest in providing explanations for the role of eDNA in biofilm matrix formation and how it is assembled and organised. For example, Holliday Junction recombination intermediates were recently proposed to contribute to the structural integrity of eDNA in biofilms^22^. Moreover, the organisation of nucleic acids into higher-order structures has implications for its rheology^23^, which is of clinical relevance for *Pseudomonas* biofilms as eDNA can be the foundation structure of a viscoelastic matrix^15,24^. Hence, resolving higher-order eDNA structures is key to understanding how and why DNA transforms from the chromosomal form to that found in the biofilm matrix.

In this study, we elucidate molecular interactions that lead to the higher-order structure of eDNA. We sought to contrast the biophysical properties of eDNA and cDNA, and correlate differences to a distinct eDNA molecular signature. The interactions that underpin eDNA’s ability to form viscoelastic structures were described by isolating eDNA while preserving its higher-order structure. This enabled the finding that guanine bases associating through non-canonical (i.e. Hoogsteen) hydrogen bonding into square planar structures, or G-quadruplex structures, rather than cDNA, is a distinctive trait of the *P. aeruginosa* biofilm extracellular matrix. G-quadruplex structures can self-assemble into higher-order viscoelastic networks^25^, and we demonstrate the interplay between G-quadruplex structures and the stability of the eDNA fibre networks in the biofilm.

## Results

### eDNA elasticity is preserved during extraction from static *P. aeruginosa* biofilms

**Fig. 1:**
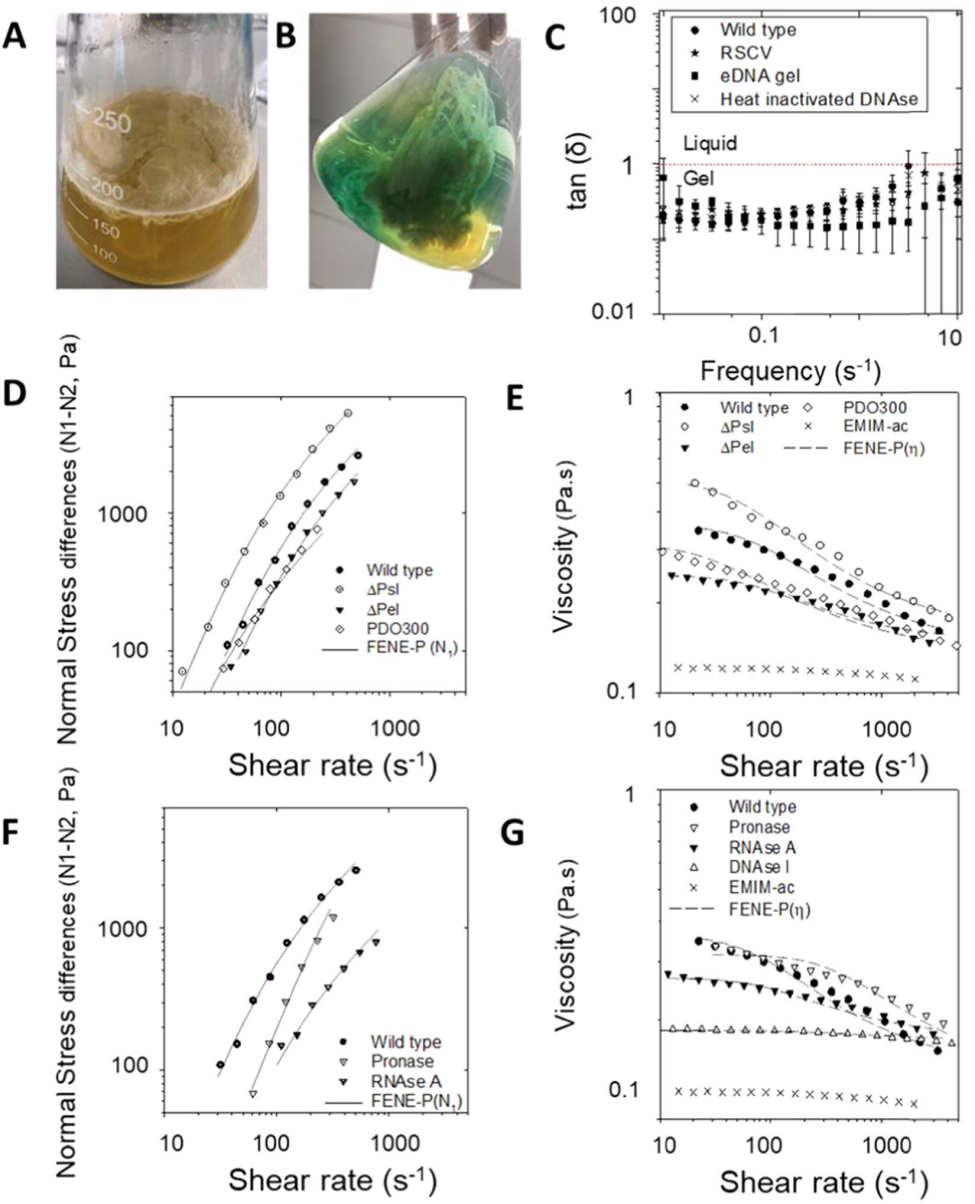
eDNA is key to *Pseudomonas aeruginosa* biofilm elasticity, pre and post dissolution in ionic liquid. Photographs of *P. aeruginosa* **A** rugose small colony variant (RSCV) pellicle (22 °C, 3 days) and **B** wild-type liquid culture (37 °C, 3 days) biofilms grown under static conditions in 500 mL conical flasks, showing exopolymer-mediated phase separation of biomass, at the surface of and in the liquid of the growth medium, respectively. **C** Frequency dependence rheogram (*n* = 2, where *n* is the number of biological replicates) showing that tan (*δ*) for wild-type, RSCV pellicle static biofilms, isolated eDNA gel and wild-type static biofilm digested with heat-inactivated DNaseI at 22 °C (250 μm plate gap) is <1 across the frequency range (red dashed line), indicating that all are viscoelastic gels. Tan (*δ*) could not be measured for DNaseI-treated biofilms as they were not in the viscoelastic region. Error bars represent the standard deviation. **D** Non-linear elasticity as representative (*n* = 2) normal stress difference (Δ*N* = *N*_1_ *−* *N*_2_) and **E** viscosity dependencies on shear rate, for *P. aeruginosa* biofilm wild type, PDO300, Δ*psl* and Δ*pel*, biofilm in 1-ethyl-3-methylimidazolium acetate (40 mg/mL) at 25 °C with 250 µm gap. **F** Representative (*n* = 2) normal stress difference (Δ*N* = *N*_1_ − *N*_2_) and **G** viscosity against shear rate for *P. aeruginosa* biofilm wild type, pronase, RNase and DNaseI-digested wild-type biofilm in 1-ethyl-3-methylimidazolium acetate (40 mg/mL) at 25 °C with 250 µm gap. Normal force is measured as a function of shear stress from 10 to 1000 Pa. Δ*N* is not described for DNaseI-digested biofilm in **F** and Supplementary Figure 1B, as their normal force (*F*_N_) is less than the resolution of the rheometer (i.e. 0.1 N) and set to zero for calculating Δ*N*. Both the Δ*N* and viscosity data are fitted with the FENE-P model, a rigid dumbbell model for polymer solutions. Fitting parameters are shown in Supplementary Table 2.

*Pseudomonas aeruginosa* biofilms formed under static conditions were selected as model systems for studying eDNA higher-order structure. eDNA provides structural features to such biofilms^26^, and static biofilms are more conducive to upscaling biofilm yield. The *P. aeruginosa* rugose small colony variant (RSCV) (i.e. 5 days) and wild-type (i.e. 5 days) strains, grown at 22 and 37 °C respectively, produced large amounts of phase-separated aggregates at the surface (Fig. 1A) and throughout the medium (Fig. 1B). However, this was not the case when RSCV was grown at 37 °C and the wild type was grown at 22 °C. Hence, the *P. aeruginosa* strains were then grown at the temperatures of increased biofilm growth, and the biofilms were harvested for in situ and ex situ studies of their eDNA.

The structural importance of eDNA was demonstrated for the static biofilms by the loss of viscoelasticity (i.e. both viscous and elastic properties) following DNaseI digestion. Under conditions of oscillating shear, tan (*δ*), which is the ratio of viscous-to-elastic response for viscoelastic materials, was <1 across the frequency range 0.1–1/s, consistent with the biofilms being classified as viscoelastic gels (Fig. 1C). An elastic response could not be measured following DNaseI digestion of the wild-type biofilm. Further, biofilms digested with heat-inactivated DNaseI remained as a gel, illustrating that the lack of measurable elastic response was due to enzymatic activity and that eDNA is an important structural component in both biofilms.

To obtain eDNA with the same viscoelastic behaviours as displayed by the biofilms, we employed ionic liquid 1-ethyl-3-methylimidazolium acetate (EMIM-Ac) to extract the matrix-associated nucleic acids^27^. The EMIM-Ac extract following biofilm dissolution displayed viscoelastic behaviour, as illustrated by the exerted normal force (i.e. contact force perpendicular to the parallel plates applying shear) and recorded viscosity, in a parallel plate rheometer. These were both measured as a function of shear rate under steady shear conditions (Fig. 1D and E, respectively). Normal force is characterised as a normal stress difference (Δ*N* = *N*_1_ *−* *N*_2_), where *N*_1_ and *N*_2_ are the primary and secondary normal stress differences, respectively (see definition in ‘Materials and methods’^28^). The fluid’s elasticity dominates the viscous flow properties for the wild-type biofilm matrix extracted by EMIM-Ac, whereby Δ*N* is an order of magnitude greater than shear stress (Fig. 1D, wild type). There is only a slight decrease in viscosity with increasing shear (i.e. shear thinning; Fig. 1E, Supplementary Figure 1A and Supplementary Table 1), which would be expected from dilute polymer solutions in viscous fluids (i.e. Boger fluids)^29^ and is consistent with other descriptions of *P. aeruginosa* biofilms^30^. The solvent (EMIM-Ac) alone exhibited no elasticity, indicating that the elastic properties arise from the dissolution of polymeric components within the biofilm matrix. Δ*N* has a power-law dependency on the shear rate with an exponent (*p*) of 1.4 (Supplementary Figure 1B, wild type), demonstrating that it is a semi-flexible polymer. The viscosity and non-linear elasticity measurements were accurately modelled using the modified, finitely extensible non-linear elastic-Peterlin (FENE-P) polymer model (line fitting Fig. 1D–G and Supplementary Tables 1 and 2), which is commonly used to describe solutions containing flexible/semi-flexible polymers^29^. As DNA is a high MW semi-flexible polymer, we hypothesised that this is the dominant polymer in solution.

Alginate, Pel and Psl are suggested structural scaffolds of *P. aeruginosa* biofilms^10^. To investigate whether the viscoelasticity of DNA during extraction by EMIM-Ac from *P. aeruginosa* biofilms relies on Pel and Psl, as described by Chew et al.^24^, we used static biofilms of alginate over-expressing strain PDO300 (i.e. isogenic mucoid deletion mutant of *P. aeruginosa* PAO1 that overproduces alginate) and its isogenic Pel and Psl genetic knockout mutants^31^. After dissolution in EMIM-Ac, when Psl was absent, the elasticity (i.e. Δ*N*) and viscosity increased relative to the wild type, and the mucoid strain PDO300 was less elastic than the wild type, despite the over-expression of alginate (Fig. 1D; Δ*psl*). In contrast, when Pel was absent, there was a slight decrease in both elasticity and viscosity relative to both the wild type and PDO300 (Fig. 1D; Δ*pel*). Nonetheless, a viscoelastic response characteristic of semi-flexible DNA was recorded without Pel or Psl present.

The contributions of proteins, RNA and DNA to the biofilm’s viscoelastic properties were also explored by digesting a static biofilm of *P. aeruginosa* wild type using pronase, RNaseA and DNaseI, respectively (Fig. 1F, G; Pronase, RNase and DNase). While removing protein and RNA individually decreased the biofilm elasticity of EMIM-Ac extracts, elasticity was maintained in both cases and all treatments displayed *p* values of 0.9–1.7 (Supplementary Table 1), characteristic of elasticity imparted by DNA^32^. In contrast, DNase treatment reduced Δ*N* to zero (result not recorded in Fig. 1F due to log scale used for Δ*N*).

**Fig. 2:**
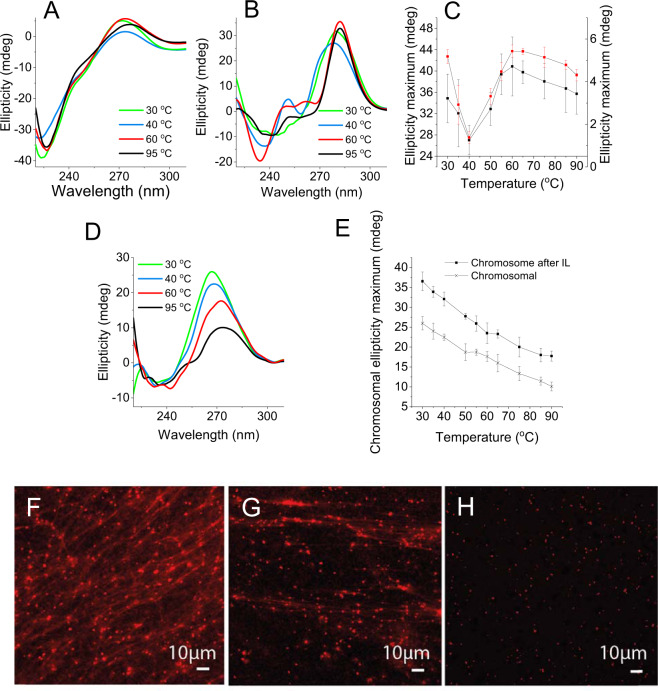
Biophysical signatures of nucleic acids are consistent with those of the eDNA fibres and are preserved when isolated. Representative circular dichroism (CD) spectra (*n* = 2) of **A** *P. aeruginosa* biofilm and **B** eDNA gel isolate at 30 °C (green), 40 °C (blue), 60 °C (red) and 95 °C (black). **C** Rolling average in amplitude (of five data points) around the mean of dominant NA peak, CD_max_ (260–285 nm), from CD spectra of *P. aeruginosa* biofilm (seen in **A**) and its extracted extracellular nucleic acid gel (i.e. isolate; seen in **B**) from *T* = 30 to 95 °C. Error bars refer to the standard deviation of the rolling averages. **D** Representative CD spectra of *P. aeruginosa* biofilm cDNA at 30 °C (green), 40 °C (blue), 60 °C (red) and 95 °C (black). **E** Rolling average in amplitude (of five data points) around the mean amplitude of dominant NA peak, CD_max_ (260–285 nm), from CD spectra of *P. aeruginosa* biofilm cDNA (seen in **D**) from *T* = 30 to 95 °C with and without solubilisation in 1-ethyl-3-methylimidazolium and fractional precipitation. Error bars refer to the standard deviation of the rolling averages. Confocal micrograph of 3-day static *P. aeruginosa* wild-type biofilm with no pre-heating (**F**), with pre-heating to 60 °C (**G**) and pre-heating to 70 °C (**H**) showing eDNA fibres in the matrix of the biofilm, illustrated by binding to DNA-specific dye propidium iodide (red), that partially and completely disappear upon heating to 60 and 70 °C, respectively. Scale bars represent 10 µm.

While there was a slight decrease in elasticity following pronase treatment, viscosity was unchanged. As with elasticity, the greatest change in viscosity was recorded after treating the biofilm with DNaseI. Thus, DNA and not proteins or the polysaccharides Pel and Psl are primarily associated with the viscoelastic properties of the biofilm matrix. The presence of DNA fibres in the extracellular matrix are exemplified in the micrograph of a static biofilm stained with TOTO-1 for DNA visualisation (Fig. 2A). The viscoelastic response upon dissolution indicates that the elasticity of eDNA is preserved upon extraction and that the ionic liquid method non-destructively extracts matrix constituents from the biofilm. The absence of phospholipids and lipopolysaccharides in EMIM-Ac following biofilm dissolution demonstrated that EMIM-Ac did not lyse either biofilm or planktonic cells (Supplementary Figure 2A).

### Key biophysical signatures of nucleic acids are preserved during isolation

We recovered the eDNA from EMIM-Ac following the dissolution of a static wild-type biofilm by exploiting the ability of perchloric acid to preferentially precipitate DNA over protein (Supplementary Figure 2B). Following further purification by gel permeation chromatography, the polymer phase separated into a gel upon transfer from EMIM-Ac into water (i.e. the gel isolate), indicating that the eDNA forms polymer networks (Supplementary Figure 3A). Calf thymus DNA did not form gels when processed the same way, either with or without added cations (Supplementary Figure 3B), demonstrating that standard (double-stranded and naked) DNA does not network and form gels under the conditions applied in this study. By contrast, for the eDNA gel, the elastic rather than the viscous response predominated (tan (*δ*) < 1) across the same frequency range as the hydrated, wild-type *P. aeruginosa* biofilms (Fig. 1C). The rheology described for the *P. aeruginosa* biofilms and eDNA gel isolate is consistent with gel behaviour, rather than the viscous properties (tan (*δ*) > 1) commonly displayed by aqueous solutions of DNA^33^. Furthermore, DNase degraded the isolated gel into shorter DNA fragments (Supplementary Figure 3C). Other fractions, including those not precipitated by perchloric acid, did not self-assemble into gels (Supplementary Figure 3D).

Circular dichroism (CD) is a highly sensitive spectroscopic technique for determining the secondary structure of biomolecules, particularly proteins and nucleic acids. It was applied here to demonstrate whether the higher-order structure of its eDNA was preserved during extraction and isolation. Unprocessed *P. aeruginosa* biofilms displayed a CD peak at 250–285 nm (Fig. 2A), which is consistent with the presence of nucleic acids^34^, and this peak dominated the CD spectrum of the gel isolate (Fig. 2B). Due to the complexity of the biofilm matrix, it is not possible to normalise CD, or ellipticity, with concentration. Therefore, scales in Fig. 2A, B are not directly comparable. Nonetheless, it can be observed from changes in the heating curves (Fig. 2C) that the nucleic acid peak amplitudes, that is, relative to the initial peak amplitudes at *T* = 30 °C, follow the same trend across the temperature profiles, with a transition from 40 to 60 °C. Only representative spectra are presented here to avoid losing structural information through data averaging. Nevertheless, nucleic acid peak transitions for eDNA were observed at the same temperature in duplicate analyses (Supplementary Figure 4). In contrast, the nucleic acid peak in cDNA extracted from *P. aeruginosa* planktonic cells decayed steadily with increasing temperature (Fig. 2D, E). This was also observed for cDNA processed through the ionic liquid (i.e. EMIM-Ac dissolution and fractional precipitation).

The change in conformation of the nucleic acids was seen to occur coincident with loss of eDNA fibres in the wild-type biofilm, which disappeared when heated to above the DNA transition temperature, while still remaining below the melting temperature of *P. aeruginosa* cDNA (i.e. ≈81 °C)^35^. As shown in Fig. 2F, G, the eDNA fibre area per image was reduced from 5263 ± 1162 at 37 °C to 2756 ± 1148 µm^2^ at 60 °C across three 3-D images (i.e. 47% reduction), and to 128 ± 26 µm^2^ at 70 °C (i.e. 98% total reduction).

Hence, heating disrupts the conformation of the nucleic acids leading to a loss of network fibre structure. Understanding the nature of this conformational change will elucidate the relationship between eDNA structure and biofilm viscoelasticity.

### Non-canonical base pairs or tetrads support extracellular network

Sequence analysis of the extracted material showed that the gene coverage was even for both cDNA and eDNA, with the exception of bacteriophage Pf4 genes (Supplementary Figure 5). While the Pf4 phage contributes to liquid crystal-like organisation of the biofilm matrix^36^, here the Pf4 knockout mutant of *P. aeruginosa* also displayed an elastic response when dissolved in EMIM-Ac (Supplementary Figure 6 and Supplementary Tables 1 and 2), demonstrating that Pf4 DNA is not responsible for the phase-separating behaviour of *P. aeruginosa* biofilms formed under these conditions.

To describe the nature of nucleic acid base-pair interactions contributing to the elastic behaviour of the isolated eDNA, we performed magic-angle spinning (MAS) solid-state nuclear magnetic resonance (SSNMR). This technique eliminates solubility and extraction biases^37^, and allows for analysis of intermolecular H-bond interactions in gels such as biofilms. MAS SSNMR averages anisotropic interactions to provide high-resolution spectral characterisation of insoluble and large biomolecular systems. Through-space heteronuclear correlations (HETCOR; e.g. N⋯H) can then be detected by analysing dipolar interactions. ^15^N–^1^H HETCOR experiments detect correlations between 1H and 15N pairs that are spatially close, such as those bonded covalently or through H bonds, with signal intensity dependent on internuclear distance and relative abundances of the atoms.

**Fig. 3:**
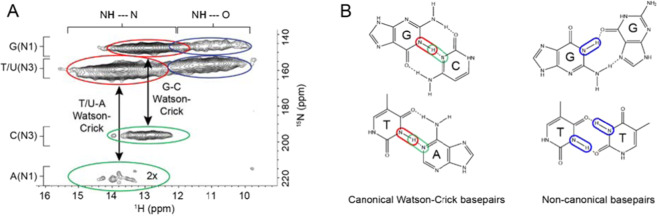
Solid-state NMR reveals the presence of non-canonical base pairs or tetrads in eDNA. **A** Representative solid-state 2D ^1^H–^15^N through-space heteronuclear correlation (HETCOR) spectrum of extracellular nucleic acid (NA) gel isolate in double-distilled water (2 mg), *T* = 25 °C. Red ovals show the cross-peaks from direct N–H couplings of G (N1–H1) and T/U (N3–H3) when the protons are hydrogen-bonded to nucleobase nitrogen atoms (i.e. NH···N). Green ovals indicate the cross-peaks generated from indirect (via hydrogen bonds) N–H couplings between G(H1) and C(N3) as well as between T/U(H3) and A(N1). The threshold for the A(N1)–T/U(H3) correlation is increased due to lower signal intensity for that particular coupling. Cross-peaks denoted by red and green ovals indicate Watson–Crick G–C and U/T–A base pairings, respectively. Blue ovals show cross-peaks from direct N–H couplings of G (N1–H1) and T/U (N3–H3) when the protons are hydrogen-bonded to carbonyl oxygen (i.e. NH···O). These cross-peaks reveal non-canonical base pairings between G, T or U and G, T, U or C as well as the possibility of G-tetrad formations. **B** Canonical and non-canonical base pairing schematics illustrate the possible correlations observed in the HETCOR spectrum. The correlations are colour-coded to match the spectrum. Covalent and hydrogen bonds are indicated by solid and dashed lines, respectively.

To elucidate the mechanism of DNA gelation, we used SSNMR to generate a 2-D, through-space, ^15^N–^1^H HETCOR spectrum of ^15^N-labelled eDNA gel isolated from *P. aeruginosa* biofilm matrix. The absence of signature protein and polysaccharide peaks in the ^15^N–^1^H HETCOR spectrum confirmed the absence of proteins and polysaccharides in the gel isolate (Supplementary Figure 7). The HETCOR spectrum across the imino proton region showed four signal clusters at *δ*_H_ 10–15 p.p.m., and *δ*_N_ 140–160 p.p.m. (Fig. 3), which arose from direct N–H couplings of T/U and G nucleobase imino groups. Two of these clusters (*δ*_H_ 12–15 p.p.m.) resulted from imino protons hydrogen-bonded to a nucleobase nitrogen (i.e. N–H⋯N), and the other two (*δ*_H_ 10–12 p.p.m) from imino protons hydrogen-bonded to a nucleobase carbonyl oxygen (i.e. N–H⋯O)^38^. Due to the longer cross-polarisation times, we were able to observe a strong, long-range and indirect (i.e. intermolecular) correlation at *δ*_N_ 196 p.p.m. arising from G–C Watson–Crick base pairing, that is, C(N3)–G(H1). There was also a weak and indirect correlation at *δ*_N_ 220 p.p.m. resulting from A–T/U Watson–Crick base pairing, that is, A(N1)–U/T(H1).

The observation of NH-to-O interactions suggests the occurrence of non-canonical base pairs, triads or tetrads involving G and T/U. The absence of long-range correlations between the clusters at *δ*_H_ 10–12 p.p.m. is also consistent with non-Watson–Crick (i.e. Hoogsteen) pairings for the G and T/U nucleobases. *δ*_H_ assignments of imino protons H-bonded to nucleobase carbonyl O and N, respectively, are validated by 1-D ^1^H NMR spectra of well-characterised Hoogsteen base-paired DNA (i.e. G-quadruplex) and canonical B-DNA structures (Supplementary Figure 8A, B), and of a DNA quadruplex–duplex structural hybrid (Supplementary Figure 8C).

**Fig. 4:**
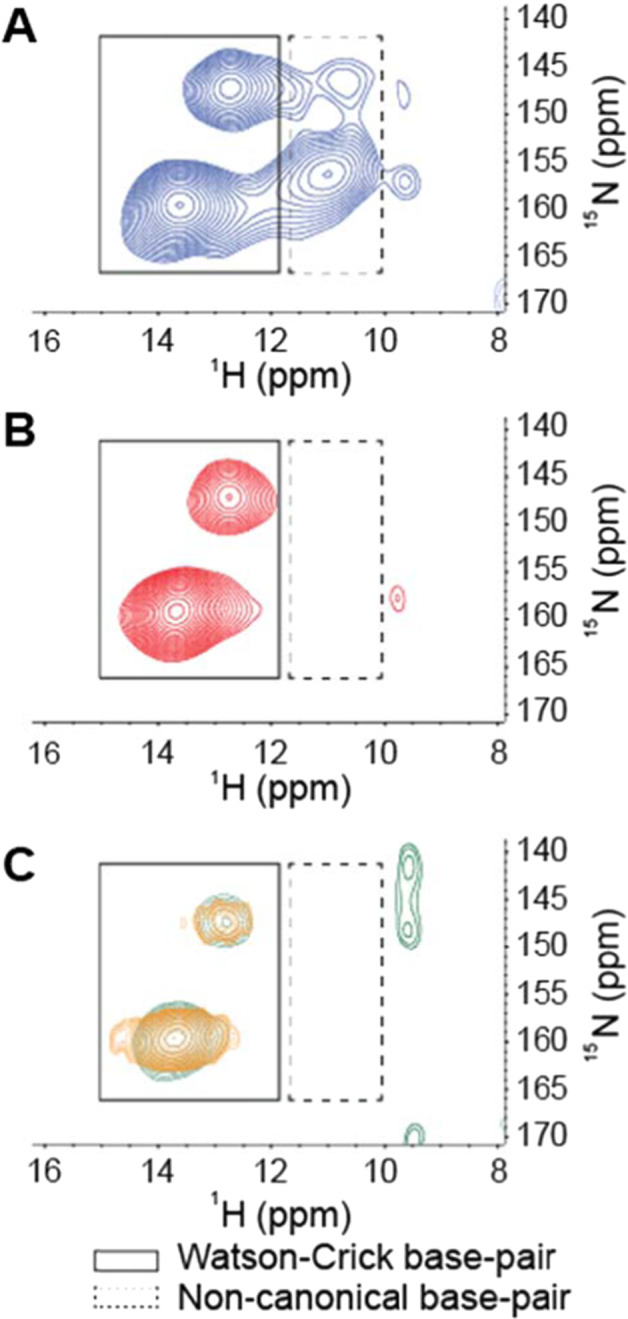
Non-canonical base pairs are a feature of networked eDNA. Representative solid-state 2D ^1^H–^15^N through-space heteronuclear correlation (HETCOR) spectrum of ^15^N-labelled eDNA gel isolate in double-distilled water (2 mg), including direct N–H coupling region only, at *T* = 25 °C with no pre-heating (**A**) and with pre-heating to 65 °C (**B**). ^15^N-labelled cDNA extracted from *P. aeruginosa* biofilms in double-distilled water before (green) and after (gold) receiving the same treatment as the eDNA (**C**), indicating the loss of non-canonical base pairings, triads or tetrads in the gel isolate upon heating and their absence in cDNA. Solid outlines indicate Watson–Crick base pairing and dotted outlines indicate non-canonical base pairing.

To determine whether non-canonical base pairs contribute to eDNA elasticity, a ^15^N–^1^H HETCOR spectrum was recorded for ^15^N-labelled eDNA gels after heating them to above the gel-sol transition temperature. In contrast to the sample without heating (Fig. 4A), no non-canonical base-pair ^15^N–^1^H interactions were detected in the sample heated to 65 °C (Fig. 4B). The Watson–Crick base-pair interactions, on the other hand, persisted with and without heating. Similarly, the cDNA of *P. aeruginosa*, which does not exhibit the temperature-dependent transition characteristic of the eDNA (Fig. 2D), displayed no NH-to-O interactions, which is consistent with the absence of non-canonical base-pair interactions (Fig. 4C). The only canonical base-pair interactions were observed in the ^15^N–^1^H HETCOR solid-state spectrum of *P. aeruginosa* cDNA before (Fig. 4C: green) and after (Fig. 4C: gold) receiving the same treatment as eDNA, demonstrating that non-canonical base-pair interactions are specific for the eDNA of *P. aeruginosa* and not an artefact of extraction and processing.

### G-quadruplex structures observed in the extracellular matrix of static and flow-cell *Pseudomonas* biofilms

**Fig. 5:**
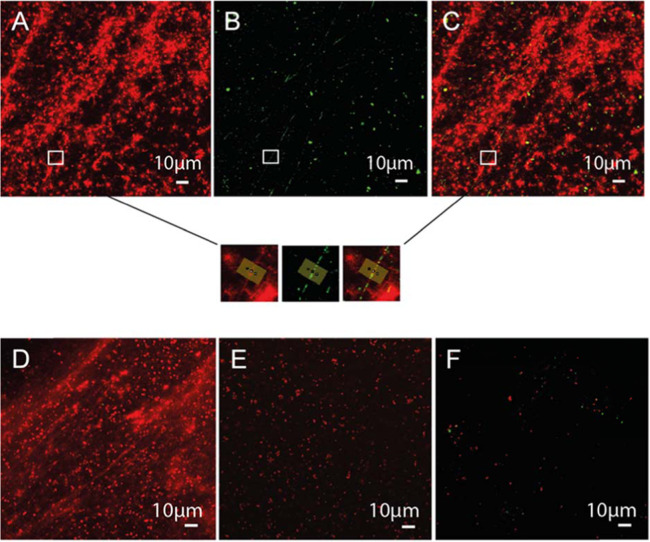
G-quadruplex DNA observed in *P. aeruginosa* biofilms and its loss coincides with the disappearance of matrix fibres. Confocal micrographs of 3-day *P. aeruginosa* rugose small colony variant (RSCV) pellicle biofilm showing the presence of eDNA fibres, visualised by binding to DNA-specific dye propidium iodide (PI; red) (**A**), the binding of anti-DNA G-quadruplex structure antibody to the eDNA fibres, visualised with GFP-labelled goat anti-mouse IgG (green) (**B**) and overlapping bindings of both PI and anti-DNA G-quadruplex antibody (**C**). The zoomed-in insets from the three images show that the anti-DNA G-quadruplex antibody binds to eDNA fibres. The horizontal line denotes the region used to describe the colocalisation of PI and G-quadruplex antibodies in terms of channel pixel intensity as a function of location, where PI and G-quadruplex colocalise at the sixth pixel of the line (Supplementary Figure 9). Confocal micrograph of the RSCV pellicle biofilm with pre-heating to 60 °C (**D**) and pre-heating to 70 °C (**E**), with PI and anti-DNA G-quadruplex antibody with GFP-labelled goat anti-mouse IgG (green) overlapping, showing almost complete disappearance of G-quadruplex structures by 60 °C, and partial and complete disappearance of eDNA fibres at 60 and 70 °C, respectively. Confocal micrograph of DNase-treated 3-day *P. aeruginosa* rugose small colony variant pellicle biofilm overlapping (PI) and anti-DNA G-quadruplex structures’ antibody with GFP-labelled goat anti-mouse IgG (green), showing that the eDNA fibres disappear coincident with the loss of anti-DNA G-quadruplex antibody binding (**F**). Scale bars represent 10 µm.

SSNMR analysis of the eDNA ex situ indicated the presence of non-canonical (i.e. Hoogsteen) triads or tetrads involving G and T/U. One possible explanation for this phenomenon is that Hoogsteen H-bonded G bases assemble planarly into tetrads and self-stack to form structurally stable G-quadruplexes^39^. Immunofluorescence confocal microscopy was thus undertaken at the surface of an undisturbed pellicle biofilm of *P. aeruginosa* RSCV (i.e. in growth chamber) exposed to G-quadruplex-specific antibody clone 1H6, which binds to DNA G-quadruplex structures, regardless of sequence, and not to non-G-quadruplex DNA^40^. This shows the coincidence of the G-quadruplex-specific antibody with eDNA fibres stained with propidium iodide (PI) (Fig. 5A–C). The colocalisation of the antibody and PI stain is further illustrated by normalised pixel intensity for the corresponding fluorescence channels along a line spanning an eDNA fibre, showing a peak in both channels at the same location (Supplementary Figure 9A). The greater incidence of PI compared to G-quadruplex antibody staining is consistent with data presented in Fig. 3A, showing a greater intensity of correlations associated with canonical rather than non-canonical interactions, and further suggests that eDNA contains a mixture of structures. Some cells were also stained by both the antibody and PI, indicating that G-quadruplex nucleic acid structures were associated with dead cells or cells with compromised membranes. As with the wild-type biofilm, DNA fibres disappear for the RSCV pellicle biofilm when heated >60 °C (i.e. the DNA transition temperature described in Fig. 2). Fibre area per image was reduced from 4112 ± 1100 to 1526 ± 673 µm^2^, or 63%, across three 3-D images, coincident with almost complete disappearance of the G-quadruplex signal (Fig. 5D). This is consistent with what was described for the eDNA gel isolate upon heating to above the DNA transition temperature determined using SSNMR (Fig. 4). All eDNA fibres disappeared by 70 °C (Fig. 5E). PI staining indicates that some DNA persisted after digestion of the pellicle biofilm by DNaseI regardless of the dilution effect of the added digestion buffer (i.e. ×1.2), and, importantly, eDNA fibres disappeared (Fig. 5D–F), which supports the loss of elasticity described in Fig. 1C.

In contrast, *P. aeruginosa* wild-type planktonic cells showed only a low incidence of extracellular binding to the antibody (Supplementary Figure 9B). cDNA displayed a very weak signal (Supplementary Figure 10A), in contrast to a well-characterised G-quadruplex structure^41^ (Supplementary Figure 10B), demonstrating that the G-quadruplex antibody is not randomly binding to DNA and that G-quadruplexes are distributed throughout the phase-separating matrix material. As an additional control, we showed that a secondary antibody bound weakly to pellicle biofilms in the absence of the G-quadruplex antibody (Supplementary Figure 11).

**Fig. 6:**
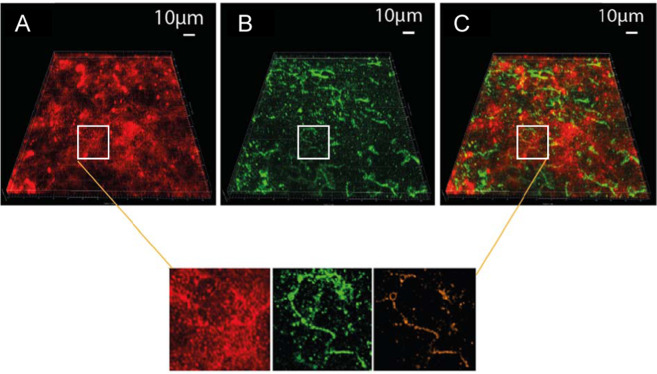
G-quadruplex DNA present in static and flow-cell *P. aeruginosa* biofilms. Three-dimensional (3-D) confocal micrographs of 3-day *P. aeruginosa* wild-type flow-cell biofilm showing the colocalisation of eDNA and G-quadruplex nucleic acid structures in the matrix of the biofilm, as illustrated by binding to DNA-specific dye propidium iodide (PI; red), (**A**) the binding of anti-DNA G-quadruplex structure antibody to the eDNA fibres, visualised with GFP-labelled goat anti-mouse IgG (green) (**B**) and overlapping binding of both PI and anti-DNA G-quadruplex antibody (**C**). The zoomed-in insets from the three images showing a region of colocalisation of G-quadruplex antibody with PI.

This binding of G-quadruplex-specific antibody to fibres in the matrix structures and some cells was also observed in a three-day flow-cell biofilm of *P. aeruginosa* wild type (see Fig. 6A–C for representative images). Fibres bound to G-quadruplex-specific antibodies were a predominant characteristic of the biofilm. The Mander’s coefficient for colocalisation of PI to G-quadruplex antibody for six z-stack images was 16 ± 6%, and there were fibre-like structures in many regions of the biofilm where both are clearly colocalised (Fig. 6, insets).

Unlike the positive control, both static and flow-cell biofilms (Figs. 5 and 6) had regions bound to the G-quadruplex antibody but not PI. The antibody specificity to G-quadruplex structures was demonstrated by the lack of binding to cDNA and cytoplasmic material of planktonic cells, as well as no binding of the secondary antibody for the matrix of the *P. aeruginosa* wild-type flow-cell biofilm (Supplementary Figure 12). The positive binding of G-quadruplex antibody to *P. aeruginosa* biofilms, therefore, confirms the presence of G-quadruplex DNA structures in its extracellular matrix.

## Discussion

Here, we address the question of how eDNA is assembled in the extracellular matrix of biofilms by describing atomic-level interactions between the nucleic acids using SSNMR. We extracted and purified the eDNA and demonstrated the fidelity of restoration by describing the biophysical signatures that eDNA shares with the biofilm, specifically with regards to temperature dependence of DNA transitions. The extracted eDNA gel isolate is thus an accurate proxy for understanding the intermolecular interactions that stabilise the extracellular matrices of *P. aeruginosa* biofilms. Observations regarding the nature of base-pair interactions by SSNMR analysis on the extracted biofilm were additionally validated by follow-up of immunofluorescence microscopy to support the interpretation that G-quadruplex eDNA is present in the biofilm matrix as well as the eDNA extract and is therefore not an artefact of processing. SSNMR analysis of the gel revealed that, as with its behaviour in EMIM-Ac, *P. aeruginosa* eDNA can self-assemble into viscoelastic networks in water without the presence of polysaccharides (e.g. Pel) or proteins. This finding contrasts with previous reports describing their colocalisation with eDNA fibre networks^17,21^.

Significantly, by through-space HETCORs (e.g. N⋯H) in the extracted eDNA matrix, we discerned the presence of non-canonical base pairs, triads or tetrads involving G and T/U, which are absent in the cDNA. Thus, *P. aeruginosa* eDNA has a different higher-order structure to its cDNA counterpart. We reasoned that cell lysis did not contribute to the elastic behaviours described, as no cell membrane components were observed in the extract, indicating that the membrane was not disturbed (Supplementary Figure 2A). Hence, we conclude that the ability to form non-canonical base pairs, triads or tetrads is a characteristic of *P. aeruginosa* biofilm eDNA.

The presence of non-canonical G base pairs strongly suggests the presence of G-quadruplex structures, and we provide further evidence supporting this by means of immunofluorescence microscopy showing binding of the matrix, of both static and flow-cell *P aeruginosa* biofilms, to a G-quadruplex-specific antibody. Moreover, we describe the loss of G-quadruplex antibody binding to a static biofilm of *P. aeruginosa* pre-heated to above the nucleic acid transition temperature, which occurred coincident with loss of eDNA network structures. The conformation change, occurring at 40–60 °C, is described using the three independent methods of microscopy, SSNMR and CD (Figs. 2 and 4A, B). Polymeric networking is a requirement for viscoelastic gels to form^42^. An additional 92% loss of eDNA fibres was observed upon heating to 70 °C, which occurred without DNA structural or conformation transition (Fig. 2B), indicating that the loss of G-quadruplexes weakened the eDNA fibres. Moreover, these observations of G-quadruplex DNA in unprocessed biofilms, responding in the same way to temperature, validates our assertion that their appearance in the ex situ eDNA was not an artefact of processing.

While the present study provides the first account of a structural signature for eDNA, which is not present in the cDNA, it is possible that G-quadruplex eDNA structures, as described here for *P. aeruginosa*, are phenomena more broadly found in other biofilm systems. In addition to *Pseudomonas* spp., other microorganisms are known to have matrix eDNA, including the clinically relevant bacteria *Staphylococcus aureus*^43^, *Staphylococcus epidermidis*^44^ and *Mycobacterium abscessus*^45^, as well as environmental isolates such as strain F8 from the South Saskatchewan River^16^. Bockelmann et al.^16^ also observed that stable filamentous networks produced by the aquatic strain F8 were comprised of DNA. eDNA was shown to specifically contribute to viscoelastic behaviour in several biofilms additional to *P. aeruginosa*, including *S. aureus*, *S. epidermidis* and *Streptococcus mutans*. Our methodology developed to elucidate the extracellular non-canonical nucleic acid structures, if employed more broadly, will likely lead to their detection across a wider range of biofilms, particularly those displaying viscoelastic behaviour^46^. Understanding how eDNA can achieve this and underpin biophysical and other emergent properties of the biofilm, via the extracellular matrix, will further inform on the regulation and control of eDNA assembly and release from cells in environmental and clinical biofilms.

Ionic liquids non-destructively dissolve a range of recalcitrant biopolymers, including DNA^47^ and cellulose^48^, and we recently demonstrated their use in dissolving *P. aeruginosa* biofilm exopolymers^27^. Long-term stability has been reported for various biomolecules in ionic liquids, including DNA^49^, and its use here for assessing higher-order eDNA structure is informative. The eDNA displays viscoelasticity throughout processing in the biofilm, as liquid upon dissolution, and upon restoration as a post-isolation gel. This is not understood to be a common feature of DNA, and we corroborated this with parallel studies of the viscoelasticity of calf thymus and *P. aeruginosa* planktonic DNA.

Studies on biofilm matrix structure have typically used the wild-type rather than RSCV strain, as knockout strains are available for Pel and Psl polysaccharides expressed by the wild type^50^. Such approaches have improved our understanding of the contribution of these exopolymers to the fundamental ability of eDNA to form networks^17^. The viscoelastic response of eDNA in EMIM-Ac was detected in the absence of other macromolecules implicated in *P. aeruginosa* biofilm matrix formation, further suggesting that DNA is the main contributor to the non-linear elasticity of lyophilised static biofilms of *P. aeruginosa* upon dissolution in EMIM-Ac. The results also suggest that other exopolymers, in part, contribute to the rheology of the matrix material in solution, with the matrix components from the Δ*psl* mutant being the most elastic, followed by wild-type and RNaseA-treated biofilms, which were the least elastic. Also, it was previously noted that a range of *P. aeruginosa* exopolymers influences biofilm extracellular matrix crosslinking^10,24,46^. The data presented here further clarify that these exopolymers can influence eDNA viscoelasticity, suggestive of their association with eDNA. Nonetheless, eDNA displays an elastic response even in the absence of other polymers. This is a departure from the prevailing paradigm that polysaccharides are principally responsible for the elastic response of biofilms. We demonstrate that the hypothesis that eDNA in the matrix forms G-quadruplex structures is valid for native biofilms of both RSCV (Fig. 5) and the isogenic wild type (Fig. 6). We, therefore, submit that the findings described here are applicable to many PAO1 variants. The elastic and biofilm-forming properties were also observed for *Pseudomonas putida* and *Pseudomonas protegens* (Supplementary Figures 6 and 13 and Supplementary Tables 1 and 2), suggesting that eDNA is broadly important for biofilm formation in several member species of this genus.

G-quadruplexes can stack to form stable higher-order structures, including viscoelastic gels^51^ and conductive nanowires^52^. They are also resistant to nucleases and stable under a wide range of environmental conditions^53^. The contributions of G-quadruplexes to preserving telomere length in eukaryotic nucleic acids^54^, regulating DNA replication^55^ and modulating virulence in microbial pathogens^56^ are known, and this study describes for the first time their appearance in biofilm matrices. While gel-forming exopolymers have been detected in other biofilms^57^, DNA does not typically form gels in biological systems. DNA gelation is normally facilitated by branched DNA molecule formation (i.e. Y-scaffold), chemical crosslinking agents (e.g. epoxides), cationic polyelectrolytes (e.g. spermidine) to promote complexes by electrostatic interactions or physical entanglements^58^. The description of G-quadruplex structures in this study informs on how bacterial DNA, otherwise favouring linear or circular structures, can form gel structures in biofilms, and thus how eDNA assembles. While DNase has already been shown to remove *P. aeruginosa* biofilms, this is not always the case (e.g. with mature biofilms). G-quadruplex structures, therefore, provide another target for biofilm control, through an understanding of the factors that allow G-quadruplex structures to form in the eDNA but not in the cDNA. This could potentially involve G-quadruplex destabilising agents identified as our understanding of G-quadruplex DNA improves^59^.

## Methods

### Bacterial strains

A dispersal-resistant *P. aeruginosa* RSCV was spontaneously derived from *P. aeruginosa* PAO1 wild-type strain with a single-nucleotide polymorphism in *wspF* and is characterised by higher levels of cyclic-di-GMP compared to the wild type^60^. The *P. aeruginosa* PAO1 Δ*pf4* knockout mutant is a defined Pf4 chromosomal deletion mutant of the entire Pf4 prophage genome^61^. *Pseudomonas aeruginosa* PDO300, PDO300 (Δ*pel*) and PDO300 (Δ*psl*) mutant strains were used (Professor Bernd H. A. Rehm’s laboratory, Institute of Molecular Biosciences, Massey University, New Zealand^31^), where PDO300 is an isogenic *mucA* deletion mutant of *P. aeruginosa* PAO1 that overproduces alginate. Lysogeny growth medium (LB) was used for all experiments, except where through-space base-pair interactions were described by SSNMR spectroscopy where a ^15^N-labelled NH_4_Cl-supplemented M9 minimal media was used for biofilm growth. M9 consisted of 9.552 g/L Na_2_HPO_4_·2H_2_O, 4.41 g/L KH_2_PO_4,_ 1.71 g/L NaCl, 1 g/L ^15^NH_4_Cl, 0.24 g/L MgSO_4_, 0.011 g/L CaCl_2_, 2 g/L casamino acids and 0.4 g/L glucose. Pre-cultures of *P. aeruginosa* PAO1 wild-type and isogenic mutant strains were grown at 37 °C, and the RSCV 22 °C. Pre-cultures of *P. protegens* Pf-5 and *P. putida* ATCC BAA-477 and S12 strain were grown in LB at 30 °C.

### Biofilm growth assays

Static biofilms of *P. aeruginosa* wild-type and isogenic mutants were prepared by diluting 10 mL aliquots of *P. aeruginosa* planktonic pre-cultures (wild type LB, 200 r.p.m., 37 °C, OD_600_ 2.40, 16 h) with LB to an OD_600_ of 0.012 in 2-L conical flasks and incubated for 5 days under static conditions to allow cells to form phase-separating gel matrices. *Pseudomonas aeruginosa* RSCV pellicle biofilms were prepared identically but at 22 °C. The wild-type static cultures were collected by centrifugation at 10,000 × *g* for 15 min to dewater the biofilm gel and the centrate was removed by decanting. Thick pellicle biofilms of RSCV were collected from the surface using tweezers. Rheological and microscopic analyses were undertaken on both sets of native biofilms. Wild-type static biofilms were additionally lyophilised (LabConco) for further treatments (i.e. solubilisations, enzymatic digestions and eDNA isolation).

### Enzymatic digestions

For normal force measurements, 20 mg of lyophilised biofilms were resuspended in 1 mL of either (i) RNase buffer (50 mM Tris-HCl, 10 mM EDTA, pH 8) with 0.2 mg RNaseA from bovine pancreas (Sigma-Aldrich), (ii) storage buffer (10 mM NaCl, 10 mM Tris-HCl) with 0.1 mg Pronase E from *Streptomyces grisens* (Sigma-Aldrich) with 0.5% (v/v) or (iii) DNaseI buffer (100 mM Tris (pH 7.5), 25 mM MgCl_2_ and CaCl_2_) with 0.2 mg DNaseI (active and inactivated) from bovine pancreas (Sigma-Aldrich). For microscopic analysis, 500 µL of pre-grown wild-type biofilm were digested, without lyophilisation, followed by the addition of 100 µL DNaseI buffer and DNaseI to a concentration of 0.4 mg/mL. All digestions were performed with shaking at 200 r.p.m. at 37 °C for 16 h. DNaseI was inactivated with the addition of 1 mM EDTA and following heating to 85 °C in DNaseI buffer for 10 min. The suspensions were then centrifuged (10,000 × *g*, 15 min), the supernatant was discarded and the pellets of the biofilm materials were analysed further, either following lyophilisation or directly by microscopy.

### Normal force measurement

Forty milligrams per millilitre solutions of lyophilised biofilms were added to 1 mL EMIM-Ac and incubated at 55 °C for 2 h. A Haake Mars 3 (Thermo Fisher Scientific) stress-controlled rotational rheometer with Peltier-controlled element at 25 °C was used for rheological measurements. Thirty-five-millimetre-diameter parallel plate geometry was used with smooth titanium plates to measure viscosity and normal stress difference (*N*_1_ − *N*_2_), where *N*_1_ is the difference between normal stresses in the direction of shearing and those oriented perpendicular to the shear plane, and *N*_2_ the difference between normal stresses perpendicular to the shear plane and those in the neutral, traverse, direction^28^. Prior to measurement, the gap error was zeroed at 4 N and gap error calculated as previously described^62–64^. The parallel plate rheometer was used as it allows rheological characterisation of ultra-low sample volumes and permits access to high shear rates for characterisation of non-linear elasticity without measurement artefacts such as inertia (i.e. Reynolds number is low). One hundred microlitres of sample was deposited on the plates. The plates were closed to 100 µm, the sample trimmed and then allowed to sit for 5 min prior to measurement. All measurements with normal force (*F*_N_) less than the resolution of the rheometer (i.e. < 0.1 N) were set to 0 before calculation of *N*_1_ − *N*_2_ using Eqs. 9–11 and viscosity from Eqs. 3–5 in Davies and Stokes^62^ for the parallel plate geometry.

Only the linearly increasing portion of the normal stress difference curves are presented (Fig. 1D, F and Supplementary Figure 1B). Above this range, normal stress difference begins to decrease again, which may be due to elastic instabilities or associating polymers^65^. Corrections were made to *N*_1_ *−* *N*_2_ to account for inertia using Eq. 17 in Davies and Stokes^62^ and to correct for the baseline residual force in the samples. Except for the DNaseI-treated biofilm, the shear rheology for all treatments could be modelled using the FENE with Gaussian closure proposed by Peterlin (FENE-P) constitutive model by varying four parameters to fit shear viscosity and normal stress difference as a function of shear rate (Supplementary Table 2). To use this model, we made the assumption that *N*_2_ « *N*_1_, and thus Δ*N* ~ *N*_1_, as is common for polymer solutions (Davies and Stokes^62^). Fitting parameters for the FENE-P model include *λ*_1_ the relaxation time, *b* a measure of the relative extensibility of the model spring, *η*_s_ the solvent viscosity and *η*_p_ the polymer contribution to the viscosity. The FENE-P equations can be written in the following format, as shown by Bird et al.^63^:

1 $$\eta = \eta _{\mathrm{s}} + \frac{{\eta _{\mathrm{p}}}}{{\lambda _1\dot \gamma }}\left( {\left( {C_2 + C_1} \right)^{\frac{1}{3}}\; - \;\left( {C_2 - C_1} \right)^{\frac{1}{3}}} \right)$$

2 $$N_1 = \frac{{2\eta _{\mathrm{p}}}}{{\lambda _1}}\left( {\left( {C_2 + C_1} \right)^{\frac{1}{3}}\; -\; \left( {C_2 - C_1} \right)^{\frac{1}{3}}} \right)^2$$

where

3 $$C_1 = \frac{b}{4}\lambda _1\dot \gamma$$

4 $$C_2 = \left( {C_1^2 + \left( {\frac{{b + 3}}{6}} \right)^3} \right)^{1/2}$$

All measurements were performed in duplicate. For clarity, one representative data set is presented in Fig. 1D–G and Supplementary Figure 1, with the respect to the power-law and FENE-P model fit to that data set. Averaged values for the FENE-P and power-law fits, with the standard deviation across three replicates shown in Supplementary Tables 1 and 2, where averages and standard deviations reflect overall data rather than analyses of individual samples. Duplicate measurements for all high shear rheological assays are presented in Supplementary Figure 14.

For oscillatory measurements, a Haake Mars 60 (Thermo Scientific) stress-controlled rotational rheometer with Peltier-controlled element at 25 °C was used. A measure of 0.25 mL biofilm samples was collected from the solution and deposited on 35-mm-diameter parallel plate geometry with smooth titanium plates. These were closed to 250 µm, and the samples were trimmed prior to measurement. A frequency range of 0.1–1/s was used at a strain in the linear viscoelastic region for each of the samples analysed.

### Assessing cell lysis by SSNMR

SSNMR experiments were performed on an 800 MHz Bruker Avance III 1-D NMR spectroscopy with ^31^P direct detection. Spectral analyses were performed using Topspin 4.0 and NMRFAM-SPARKY^66^ software. Asolectin (Sigma-Aldrich) standard (10 mg/mL) and lyophilised *P. aeruginosa* biofilm (10 mg/mL) were dissolved in 40% (v/v) EMIM-Ac:60% (v/v) *N*,*N*-dimethyl acetamide (DMAc) at 55 °C for 2 h. *Pseudomonas aeruginosa* pre-culture cell lysate was prepared by lysing pre-culture cells with lysozyme in phosphate-buffered saline (PBS). Ten percent (v/v) of D_2_O was added to all samples for locking purposes.

### Extracellular polymeric substance extraction

Lyophilised biofilms were dissolved in ionic liquid mixture (40% (v/v) EMIM-Ac:60% (v/v) DMAc) at 55 °C for 16 h. The solution was centrifuged (10,000 × *g*) to remove any undissolved material. Perchloric acid (70%) was added (0.05% v/v) to the viscosified centrate (on ice). After 15 min incubation, the solution was centrifuged at 10,000 × *g* at 4 °C for 15 min and the pellet recovered. This was repeated on the centrate two to four times until the solution was no longer viscous. The precipitate was dialysed against double-distilled water (ddH_2_O) for 2 days at 4 °C (SnakeSkin™ Dialysis Tubing, 3.5K molecular weight cut-off (MWCO), 22 mm) and the retentate lyophilised (FreeZone Plus 4.5 Liter Cascade Benchtop Freeze Dry System). The same procedure was performed in triplicate on calf thymus DNA, lipase and cytochrome *c* to determine the recovery yield of representative exoproteins.

### eDNA isolation

Twenty milligrams of lyophilised retentate (i.e. post perchloric acid precipitation) was dissolved in 1 mL of 40% (v/v) EMIM-Ac:60% DMAc (v/v) (55 °C, 16 h). Chromatographic separation was achieved in a Shimadzu System comprising DGU-20A 3r Prominence Degasser and LC-20AD Solvent Delivery Unit, fitted with two Agilent PLgel 10 μm columns of 10^5^ Å pore size for separation across the MW range 200–2000 kDa. The eluent flow rate was 3.0 mL/min and the injection volume 1 mL. The fractions with MW range of 2000–800 and 800–200 kDa were pooled, precipitated with 60% (v/v) ethanol and the precipitate resuspended in ddH_2_O and dialysed for 2 days at 4 °C (SnakeSkin™ Dialysis Tubing, 3.5K MWCO, 22 mm) against ddH_2_O to induce gelation. The gel was then collected from the dialysis tubing.

### Temperature-dependent DNA transitions

Five-day-old *P. aeruginosa* wild-type biofilm and eDNA gel isolate were resuspended in ddH_2_O to achieve ultraviolet (UV) absorbance reading 1 and ddH_2_O served as a blank. The heat-treated samples were analysed by JASCO-815 spectropolarimeter in a 1 cm path length quartz cuvette containing a solution volume of 500 μL. Spectra (200–320 nm) were measured at 1 °C increment from 30 to 95 °C. For each measurement, an average of three scans was taken and the buffer spectra subtracted. The magnitude of the DNA peak presented is the rolling average across five temperatures. For eDNA networking visualisation assays, 1 mL aliquots of pre-grown wild-type and RSCV biofilms were added to 50 mL sterile tubes and placed in 37, 60 and 70 °C water baths for 18 h.

### Determining through-space correlations by NMR

SSNMR experiments were performed on the eDNA gel isolate, whereby the eDNA gel was prepared from the M9-cultured *P. aeruginosa* biofilm, as described above.

SSNMR experiments were performed on eDNA gel (1) with and (2) without pre-heating to 65 °C; in addition to (3) cDNA after lyophilisation, dissolution in EMIM-Ac:60% (v/v) DMAc at 55 °C for 16 h, precipitation with 60% ethanol (v/v) and dialysis against water (3500 Da MWCO), as described above for eDNA. A 14.1 T Bruker Advance III instrument was used, equipped with a 1.9 mm MAS probe operated in double mode. The typical ^1^H, ^15^N and ^31^P *π*/2 pulse lengths were 2.3, 3.7 and 4.5 μs, respectively. 2-D dipolar-based ^15^N–^1^H HETCOR experiments were conducted on the ^15^N-labelled eDNA gel isolate at 37 kHz MAS spinning frequency. The variable temperature was regulated at −20 °C and the sample temperature was 12 °C (calibrated using ethylene glycol). In the ^15^N–^1^H HETCOR experiments, the initially excited ^1^H magnetisation was transferred to ^15^N through a cross-polarisation step, followed by t1 evolution. The ^15^N magnetisation was then flipped to the longitudinal axis and 400 ms proton saturation pulses were applied for water suppression. Subsequently, the ^15^N magnetisation was flipped to the transverse plane and transferred to ^1^H via a second CP step for signal acquisition. Two ^15^N–^1^H HETCOR spectra were recorded, one with 400 μs and the other with 2 ms contact times applied for both of the CP steps. Low-power XiX ^1^H decoupling (~10 kHz) was employed during ^15^N evolution and WALTZ-16 decoupling (10 kHz) was implemented on ^15^N channel during ^1^H acquisition.

1-D ^31^P experiments were performed on ^15^N-labelled eDNA gel isolate and ^15^N-labelled *P. aeruginosa* biofilm, both directly after dialysis against ddH_2_O at 4 °C for 2 days (SnakeSkin™ Dialysis Tubing, 3.5 K MWCO, 22 mm) and following alkalinisation (0.1 M NaOD, 55 °C, 15 min) and lyophilisation (FreeZone Plus 4.5 Liter Cascade Benchtop Freeze Dry System). A MAS spinning frequency of fifteen kilohertz, sample temperature of 27 °C, and 75 kHz SPINAL64 1H decoupling, were applied. All chemical shifts were indirectly referenced using adamantane as a secondary standard (downfield peak is at 40.48 p.p.m., DSS scale).

### cDNA extraction

cDNA was extracted from the biofilm using FastDNA SPIN Kit for soil (MP Biomedicals, USA) as per the standard protocol. Briefly, biofilm was resuspended in sodium phosphate buffer for lysis (i.e. lysing matrix), homogenised (FastPrep^®^, 40 s, speed setting 6.0) and the cell debris removed by centrifugation (14,000 × *g*, 5 min). Proteins were removed by precipitation (250 μL protein precipitation solution) and the supernatant was mixed with DNA-binding matrix, which was then homogenised and transferred to a SPIN™ Filter. Excess supernatant was removed by centrifugation (14,000 × *g*, 5 min). DNA was then eluted from an air-dried, DNA-binding matrix with DNase/pyrogen-free water.

### Nucleotide sequencing and analysis

The eDNA gel isolate was resuspended in 500 uL of Protease K solution (50 mM Tris-HCl, pH 8, 1% SDS, 1 mM CaCl_2_). 10 mL of Proteinase K (Thermo Fisher Scientific) was added (final concentration 0.4 mg/mL) and the mixture was incubated at 56 °C for 16 h. DNA was then extracted as previously described in the phenol-chloroform method^67^.

Prior to sequencing, samples were further purified to remove any remaining protein and RNA by RNase and Proteinase K treatment. The DNA was then isolated using phenol–chloroform precipitation as described above. The DNA precipitate was dissolved in TE buffer, the purity confirmed by 260/280 value in Nanodrop (acceptable range value: 1.8–2.0) and Qubit^®^ 2.0 fluorometer.

The MW distributions of extracellular and genomic DNA were measured on a 1% agarose gel, which was prepared from Viviantis LE grade agarose using 1× TAE buffer (40 mM Tris, 20 mM acetate and 1 mM EDTA, pH 8.6). Gels were run horizontally. After electrophoresis, the gel was stained for 0.5 h with ethidium bromide and visualised under UV light^68^.

Three biological replicates were used for each DNA sequence analysis. Each library was produced using Illumina DNA sample preparation kit. The libraries were sequenced using Illumina MiSeq platform (Illumina, San Diego, CA) with paired-end protocol to read lengths of 600 nucleotides, generating a total of 1,614,106 and 1,848,846 paired-end reads. Raw reads were quality filtered (reads remaining after trimming: PPG1-1549104, PBLC1-1666280) and aligned to the *P. aeruginosa* PAO1 (AE004091) genome using CLC Genomics Workbench 9.0 (CLC bio, Cambridge, MA). Extracellular RNA length was determined by TapeStation, model 2200 (Agilent Technologies).

### Staining and microscopy

For the purposes of staining and imaging, *P*. *aeruginosa* RSCV pellicle biofilms were grown statically, as described above, in an eight-well chambered coverslip (Ibidi, 25 × 75 mm^2^). Pre-grown and treated wild-type biofilms were deposited (100 µL) into chambered coverslip for imaging. *Pseudomonas* *aeruginosa* wild-type flow-cell biofilms were grown in a 1 × 4 × 40 mm^3^ three-channel setup, where overnight cultures (optical density 0.5, 10^8^ cells) were injected into a flow-cell operated at a flow rate of 9 mL/h. The flow cell was initially inverted for 1 h to allow for irreversible attachment of cells to the glass slide. Biofilms were grown for 3 days and flushed twice with LB media.

All microscopic imaging was conducted on a Zeiss LSM 780 confocal microscope with a ×63 objective. eDNA staining of static biofilms and eDNA gel isolate was achieved by depositing them on a glass slide and air-dried overnight. eDNA was stained by incubating with PI (10 µM) in NaCl (0.9% w/v).

G-quadruplex DNA-specific primary antibody 1H6 (2 µg/mL; Sigma-Aldrich) in 1× PBS was introduced to chambers for 1 h, followed with flushing (9 mL/h) twice for 2 min each. Anti-DNA antibodies were detected with GFP-labelled (Alexa Fluor 488) goat anti-mouse IgG (2 mg/mL stock solution, 2 µg/mL) in 1× PBS (1 h exposure).

eDNA fibre area quantification was performed using ImageJ. An intensity threshold was used to remove the cell and background signals, which have high and low intensities, respectively. This process removed the bright cells, thus enabling visualisation of fibres. The threshold image was converted into pixel values of 0 and 1. A value of 1 indicates that the pixel contains intensity from the fibre. The pixel values of the whole image were summed and multiplied by the pixel area to give the area of fibre in µm^2^. Mander’s coefficient was calculated using the Imaris software. Line plot analysis was performed by integrating the intensity of fibres (seen as a brown horizontal line in zoomed-in image) in a 12 × 20 pixel region of interest. The intensity of both channels was normalised and plotted against the chosen pixel size.

### Reporting summary

Further information on research design is available in the Nature Research Reporting Summary linked to this article.

## Supplementary information

Supplementary Information

Reporting Summary

## Data Availability

The data that support the findings of this study are available from the corresponding author upon reasonable request.

## References

[1] Phillips PL, Schultz GS (2012). Molecular mechanisms of biofilm infection: biofilm virulence factors. Adv. Wound Care.

[2] Drescher K, Shen Y, Bassler BL, Stone HA (2013). Biofilm streamers cause catastrophic disruption of flow with consequences for environmental and medical systems. Proc. Natl Acad. Sci. USA.

[3] Seviour T, Lambert LK, Pijuan M, Yuan Z (2011). Selectively inducing the synthesis of a key structural exopolysaccharide in aerobic granules by enriching for *Candidatus* ‘Competibacter phosphatis’. Appl. Microbiol. Biotechnol..

[4] Høiby N, Bjarnsholt T, Givskov M, Molin S, Ciofu O (2010). Antibiotic resistance of bacterial biofilms. Int. J. Antimicrobial Agents.

[5] Kurniawan A, Yamamoto T, Tsuchiya Y, Morisaki H (2012). Analysis of the ion adsorption–desorption characteristics of biofilm matrices. Microbes Environ..

[6] de Kreuk MK, Picioreanu C, Hosseini M, Xavier JB, van Loosdrecht MCM (2007). Kinetic model of a granular sludge SBR: Influences on nutrient removal. Biotechnol. Bioeng..

[7] Flemming H-C, Wingender J (2010). The biofilm matrix. Nat. Rev. Microbiol..

[8] Gloag ES, Fabbri S, Wozniak DJ, Stoodley P (2020). Biofilm mechanics: Implications in infection and survival. Biofilm.

[9] Bodey GP, Bolivar R, Fainstein V, Jadeja L (1983). Infections caused by *Pseudomonas aeruginosa*. Rev. Infect. Dis..

[10] Colvin KM (2012). The Pel and Psl polysaccharides provide *Pseudomonas aeruginosa* structural redundancy within the biofilm matrix. Environ. Microbiol..

[11] Seviour T (2015). Functional amyloids keep quorum-sensing molecules in check. J. Biol. Chem..

[12] Borlee BR (2010). *Pseudomonas aeruginosa* uses a cyclic-di-GMP-regulated adhesin to reinforce the biofilm extracellular matrix. Mol. Microbiol..

[13] Allesen-Holm M (2006). A characterization of DNA release in *Pseudomonas aeruginosa* cultures and biofilms. Mol. Microbiol..

[14] Okshevsky M, Meyer RL (2015). The role of extracellular DNA in the establishment, maintenance and perpetuation of bacterial biofilms. Crit. Rev. Microbiol..

[15] Whitchurch CB, Tolker-Nielsen T, Ragas PC, Mattick JS (2002). Extracellular DNA required for bacterial biofilm formation. Science.

[16] Böckelmann U (2006). Bacterial extracellular DNA forming a defined network-like structure. FEMS Microbiol. Lett..

[17] Jennings LK (2015). Pel is a cationic exopolysaccharide that cross-links extracellular DNA in the *Pseudomonas aeruginosa* biofilm matrix. Proc. Natl Acad. Sci. USA.

[18] Cherny KE, Sauer K (2019). *Pseudomonas aeruginosa* requires the DNA-specific endonuclease EndA to degrade extracellular genomic DNA to disperse from the biofilm. J. Bacteriol..

[19] Turnbull L (2016). Explosive cell lysis as a mechanism for the biogenesis of bacterial membrane vesicles and biofilms. Nat. Commun..

[20] Niki H, Yamaichi Y, Hiraga S (2000). Dynamic organization of chromosomal DNA in *Escherichia coli*. Genes Dev..

[21] Devaraj A, Justice SS, Bakaletz LO, Goodman SD (2015). DNABII proteins play a central role in UPEC biofilm structure. Mol. Microbiol..

[22] Devaraj A (2019). The extracellular DNA lattice of bacterial biofilms is structurally related to Holliday junction recombination intermediates. Proc. Natl Acad. Sci. USA.

[23] Xing Z (2018). Microrheology of DNA hydrogels. Proc. Natl Acad. Sci. USA.

[24] Chew, S. C. et al. Dynamic remodeling of microbial biofilms by functionally distinct exopolysaccharides. *mBio* **5**, e01536-01514 (2014). 10.1128/mBio.01536-14 PMC4128364 25096883

[25] Hu J, Wang H, Hu Q, Cheng Y (2019). G-quadruplex-based antiviral hydrogels by direct gelation of clinical drugs. Mater. Chem. Front..

[26] Wang S (2015). The exopolysaccharide Psl–eDNA interaction enables the formation of a biofilm skeleton in *Pseudomonas aeruginosa*. Environ. Microbiol. Rep..

[27] Seviour T (2015). Solvent optimization for bacterial extracellular matrices: a solution for the insoluble. RSC Adv..

[28] Verrelli DI, Kilcullen AR (2016). Normal stress differences and yield stresses in attractive particle networks. Adv. Condens. Matter Phys..

[29] Stokes JR, Graham LJW, Lawson NJ, Boger DV (2001). Swirling flow of viscoelastic fluids. Part 2. Elastic effects. J. Fluid Mech..

[30] Jana S (2020). Nonlinear rheological characteristics of single species bacterial biofilms. npj Biofilms Microbiomes.

[31] Ghafoor A, Hay ID, Rehm BHA (2011). Role of exopolysaccharides in *Pseudomonas aeruginosa* biofilm formation and architecture. Appl. Environ. Microbiol..

[32] Mansfield ML, Tsortos A, Douglas JF (2015). Persistent draining crossover in DNA and other semi-flexible polymers: evidence from hydrodynamic models and extensive measurements on DNA solutions. J. Chem. Phys..

[33] Elkin I, Weight AK, Klibanov AM (2015). Markedly lowering the viscosity of aqueous solutions of DNA by additives. Int. J. Pharm..

[34] Kypr J, Kejnovská I, Renčiuk D, Vorlíčková M (2009). Circular dichroism and conformational polymorphism of DNA. Nucleic Acids Res..

[35] Tahmasebi H, Dehbashi S, Arabestani M (2020). New approach to identify colistin-resistant Pseudomonas aeruginosa by high-resolution melting curve analysis assay. Lett. Appl. Microbiol..

[36] Secor PR (2015). Filamentous bacteriophage promote biofilm assembly and function. Cell Host Microbe.

[37] Reichhardt C, Cegelski L (2014). Solid-state NMR for bacterial biofilms. Mol. Phys..

[38] Feigon, J., Koshlap, K. M. & Smith, F. W. [10]^1^H NMR spectroscopy of DNA triplexes and quadruplexes. *Methods Enzymol.* **261**, 225–255 (1995). 10.1016/s0076-6879(95)61012-x 8569497

[39] Hänsel-Hertsch R, Di Antonio M, Balasubramanian S (2017). DNA G-quadruplexes in the human genome: detection, functions and therapeutic potential. Nat. Rev. Mol. Cell Biol..

[40] Henderson A (2014). Detection of G-quadruplex DNA in mammalian cells. Nucleic Acids Res..

[41] Phan AT, Modi YS, Patel DJ (2004). Propeller-type parallel-stranded G-quadruplexes in the human c-myc promoter. J. Am. Chem. Soc..

[42] Gu, Y., Zhao, J. & Johnson, J. A. Polymer networks: from plastics and gels to porous frameworks. *Angew. Chem. Int. Ed.* **59**, 5022–5049 (2020). 10.1002/anie.201902900 31310443

[43] Mann EE (2009). Modulation of eDNA release and degradation affects *Staphylococcus aureus* biofilm maturation. PLoS ONE.

[44] Adam B, Baillie GS, Douglas LJ (2002). Mixed species biofilms of *Candida albicans* and *Staphylococcus epidermidis*. J. Med. Microbiol..

[45] Rose SJ, Babrak LM, Bermudez LE (2015). *Mycobacterium avium* possesses extracellular DNA that contributes to biofilm formation, structural integrity, and tolerance to antibiotics. PLoS ONE.

[46] Peterson, B. W. et al. Role of eDNA in the viscoelastic relaxation of biofilms. *mBio* **4**, e00497-00413 (2013). 10.1128/mBio.00497-13 PMC3812712 24129256

[47] Zhao H (2015). DNA stability in ionic liquids and deep eutectic solvents. J. Chem. Technol. Biotechnol..

[48] Vitz J, Erdmenger T, Haensch C, Schubert US (2009). Extended dissolution studies of cellulose in imidazolium based ionic liquids. Green. Chem..

[49] Singh N, Sharma M, Mondal D, Pereira MM, Prasad K (2017). Very high concentration solubility and long-term stability of DNA in an ammonium-based ionic liquid: a suitable medium for nucleic acid packaging and preservation. ACS Sustain. Chem. Eng..

[50] Ghafoor A, Hay ID, Rehm BH (2011). Role of exopolysaccharides in *Pseudomonas aeruginosa* biofilm formation and architecture. Appl. Environ. Microbiol..

[51] Bryan TM, Baumann P (2011). G-quadruplexes: from guanine gels to chemotherapeutics. Mol. Biotechnol..

[52] Bose K, Lech CJ, Heddi B, Phan AT (2018). AFM structure of DNA G-wires in aqueous solution. Nat. Commun..

[53] Maizels N (2015). G4‐associated human diseases. EMBO Rep..

[54] Moye AL (2015). Telomeric G-quadruplexes are a substrate and site of localization for human telomerase. Nat. Commun..

[55] Mirkin SM (2013). Driving past four-stranded snags. Nature.

[56] Harris LM, Merrick CJ (2015). G-quadruplexes in pathogens: a common route to virulence control?. PLoS Pathog..

[57] Seviour T, Pijuan M, Nicholson T, Keller J, Yuan Z (2009). Gel-forming exopolysaccharides explain basic differences between structures of aerobic sludge granules and floccular sludges. Water Res..

[58] Xing Y (2011). Self-assembled DNA hydrogels with designable thermal and enzymatic responsiveness. Adv. Mater..

[59] Budhathoki JB (2014). RecQ-core of BLM unfolds telomeric G-quadruplex in the absence of ATP. Nucleic Acids Res..

[60] Harikrishnan, A. S. N. *c-di-GMP Distribution Across Single and Mixed Specie*the separation of DNA fragments*s Biofilms*. Ph.D. thesis, Nanyang Technological Univ. (2016).

[61] Rice SA (2009). The biofilm life cycle and virulence of *Pseudomonas aeruginosa* are dependent on a filamentous prophage. ISME J..

[62] Davies GA, Stokes JR (2008). Thin film and high shear rheology of multiphase complex fluids. J. Non-Newton. Fluid Mech..

[63] Bird, R. B., Armstrong, R. C. & Hassager, O. *Dynamics of Polymeric Liquids* 2nd edn: Fluid mechanics, Vol. 1 (Wiley, 1987).

[64] Kravchuk O, Stokes JR (2013). Review of algorithms for estimating the gap error correction in narrow gap parallel plate rheology. J. Rheol..

[65] Annable T, Buscall R, Ettelaie R, Whittlestone D (1993). The rheology of solutions of associating polymers: comparison of experimental behavior with transient network theory. J. Rheol..

[66] Lee W, Tonelli M, Markley JL (2014). NMRFAM-SPARKY: enhanced software for biomolecular NMR spectroscopy. Bioinformatics.

[67] Ausubel, F. M. *Short Protocols in Molecular Biology: A Compendium of Methods from Current Protocols in Molecular Biology* 5th edn (Wiley, 2002).

[68] Lee PY, Costumbrado J, Hsu C-Y, Kim YH (2012). Agarose gel electrophoresis for the separation of DNA fragments. J. Vis. Exp..

