# Supplementary material for: The biofilm matrix scaffold of Pseudomonas aeruginosa contains G-quadruplex extracellular DNA structures
Source: NPJ Biofilms Microbiomes. 2021 Mar 19;7:27. doi: 10.1038/s41522-021-00197-5 (PMC7979868; doi:10.1038/s41522-021-00197-5)
Supplement: Supplementary file 2 — Reporting Summary [file 41522_2021_197_MOESM2_ESM.pdf]

Supplementary Information for

The biofilm matrix scaffold of *Pseudomonas* species contains G-quadruplex extracellular DNA structures

Thomas Seviour[1,2*], Fernaldo Richtia Winnerdy[3], Lan Li Wong[1], Xiangyan Shi[3], Sudarsan Mugunthan[1], Yong Hwee Foo[1], Remi Castaing[4], Sunil S Adav[5], Gurjeet Singh Kohli[1], Heather M Shewan[6], Jason R Stokes[6], Scott A Rice[1,7,8], Anh Tuân Phan[3], Staffan Kjelleberg[1,8,9,*]

[1] Singapore Centre for Environmental Life Sciences Engineering, Nanyang Technological University, 637551, Singapore. [2] Centre for Water Technology (WATEC), Aarhus University, Aarhus 8000, Denmark. [3] School of Physical and Mathematical Sciences, Nanyang Technological University, 637371, Singapore. [4] Materials and Chemical Characterisation Facility (MC[2]), University of Bath, BA27AY, Bath, United Kingdom. [5] Singapore Phenome Centre, Nanyang Technological University, 636921, Singapore. [6] School of Chemical Engineering, The University of Queensland, 4072, Brisbane, Australia. [7] The iThree Institute, The University of Technology Sydney, Sydney, 2007, Australia. [8] School of Biological Sciences, Nanyang Technological University, 637551, Singapore. [9] School of Biological, Earth and Environmental Sciences, University of New South Wales, Sydney, 2052, Australia.

*Correspondence to: twseviour@eng.au.dk; laskjelleberg@ntu.edu.sg

This PDF file includes:

Supplementary Figures 1 to 14
Supplementary Tables 1-2

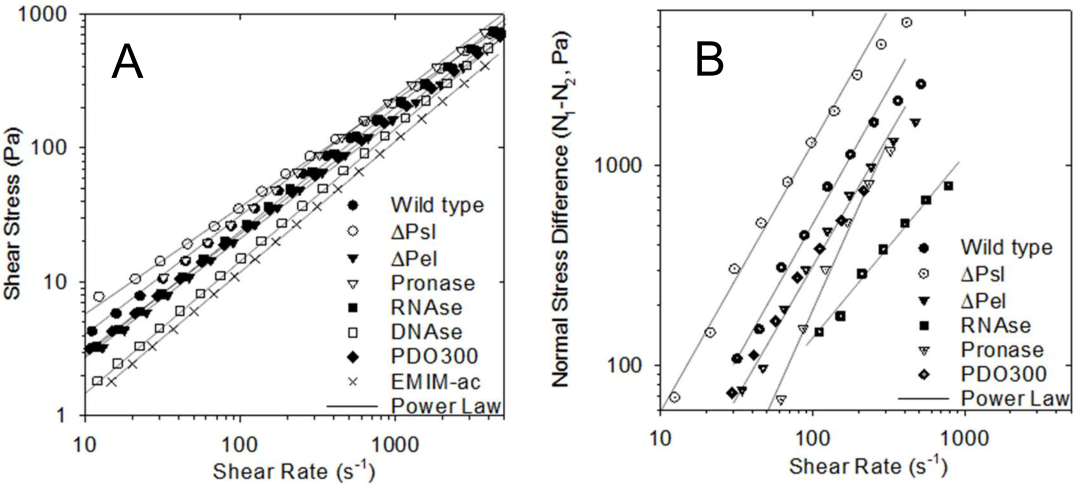

Supplementary Figure 1: (A) Shear stress and (B) normal stress differences ($N_1 - N_2$) as a function of shear rate for *P. aeruginosa* biofilms: wild type (solid circle); PDO300 (black diamond); ΔPsl (open circle), ΔPel (black triangle); pronase digested (open triangle); RNase A digested (black square); and DNase I digested wild type biofilm (open square); dissolved in 1-ethyl-3-methylimidazolium acetate (EMIM-ac; 40 mg/mL) at 25 °C (cross); with 100 µm rheometer measurement gap, and shear stress sweep from 10 to 1000 Pa. Lines indicate Power Law fits to the data. The Power Law dependences of shear stress on shear rate (m) (1C; Table S1) indicate Newtonian-like rheological properties. ($N_1 - N_2$) is not described for DNase I digested biofilm as its normal force (F_N) is less than the resolution of the rheometer (i.e., 0.1 N) and is set to zero for calculating ($N_1 - N_2$). Fitting parameters are shown in Supplementary Table 2. Representative rheograms are presented (n = 2).

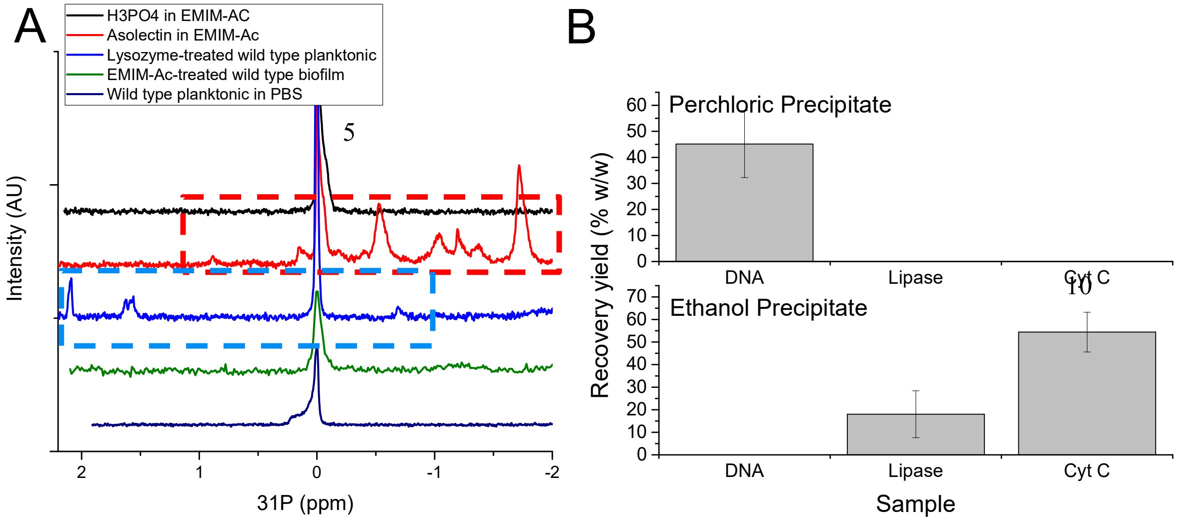

Supplementary Figure 2: (A) ^{31}P NMR spectrum of H_3PO_4 solution in EMIM-Ac (— upper), asolectin standard in EMIM-Ac (— second upper), SDS and lysozyme-treated *P. aeruginosa* wild-type planktonic cells (— middle), lyophilised *P. aeruginosa* wild type biofilm in EMIM-Ac (10 mg/mL) (— second lower) and planktonic cells in 1x PBS (— lower) at 25 °C, showing phospholipid peaks for asolectin in EMIM-Ac (red dashed box), for lysed *P. aeruginosa* cells (SDS, lysozyme) in water (blue dashed box), and the absence of phospholipid peaks in the spectrum of *P. aeruginosa* treated with EMIM-Ac. (B) Recovery yield (n = 3) of calf thymus DNA, lipase and cytochrome c standards following EMIM-Ac solubilisation and recovery with perchloric acid (upper) followed by ethanol (lower). Error bars indicate standard deviation.

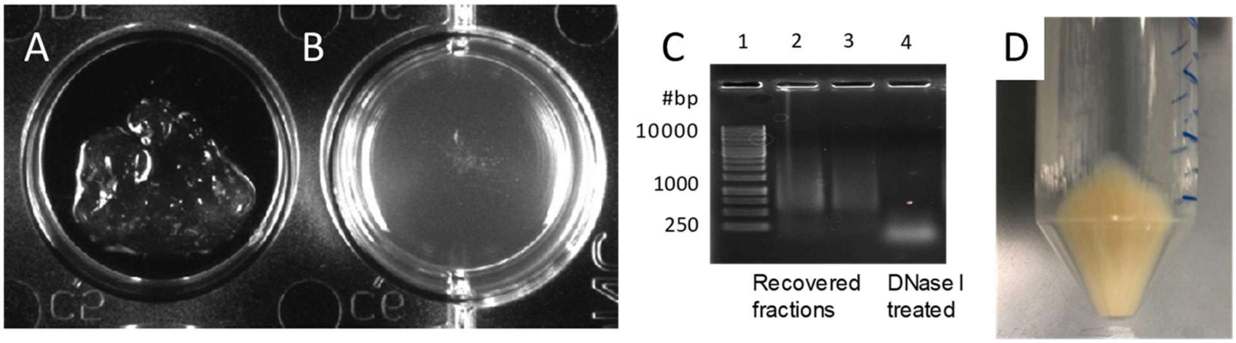

Supplementary Figure 3: (A) Photograph of eDNA extracted and purified from *P. aeruginosa* static wild type biofilm using 1-ethyl-3-imidazolium (EMIM-Ac), upon transfer in to double distilled water demonstrating that it phase-separates into a gel. (B) Photograph of calf thymus DNA at the same concentration as the eDNA gel (i.e., 26 mg/mL) processed the same way with EMIM-Ac, showing that gelation is not a universal feature of all DNA following dissolution in EMIM-Ac with perchloric acid precipitation. Both images were captured by Gel Doc^{TM} XR+ (Biorad) in a 15.6 mm plastic well. (C) Agarose electrophoretic gel of eDNA isolated from *P. aeruginosa* wild type biofilm (lanes 2 and 3) and digested with DNase (lane 4). Lane 1 is the GeneRuler 1 kbp ladder. (D) Photograph of *P. aeruginosa* biofilm extracellular polymeric substances recovered after the eDNA was removed by perchloric precipitation (see Extracellular DNA isolation in Methods and Materials) showing that gelation was not observed in the fractions without DNA. Image was captured in a 50 mL sterile tube.

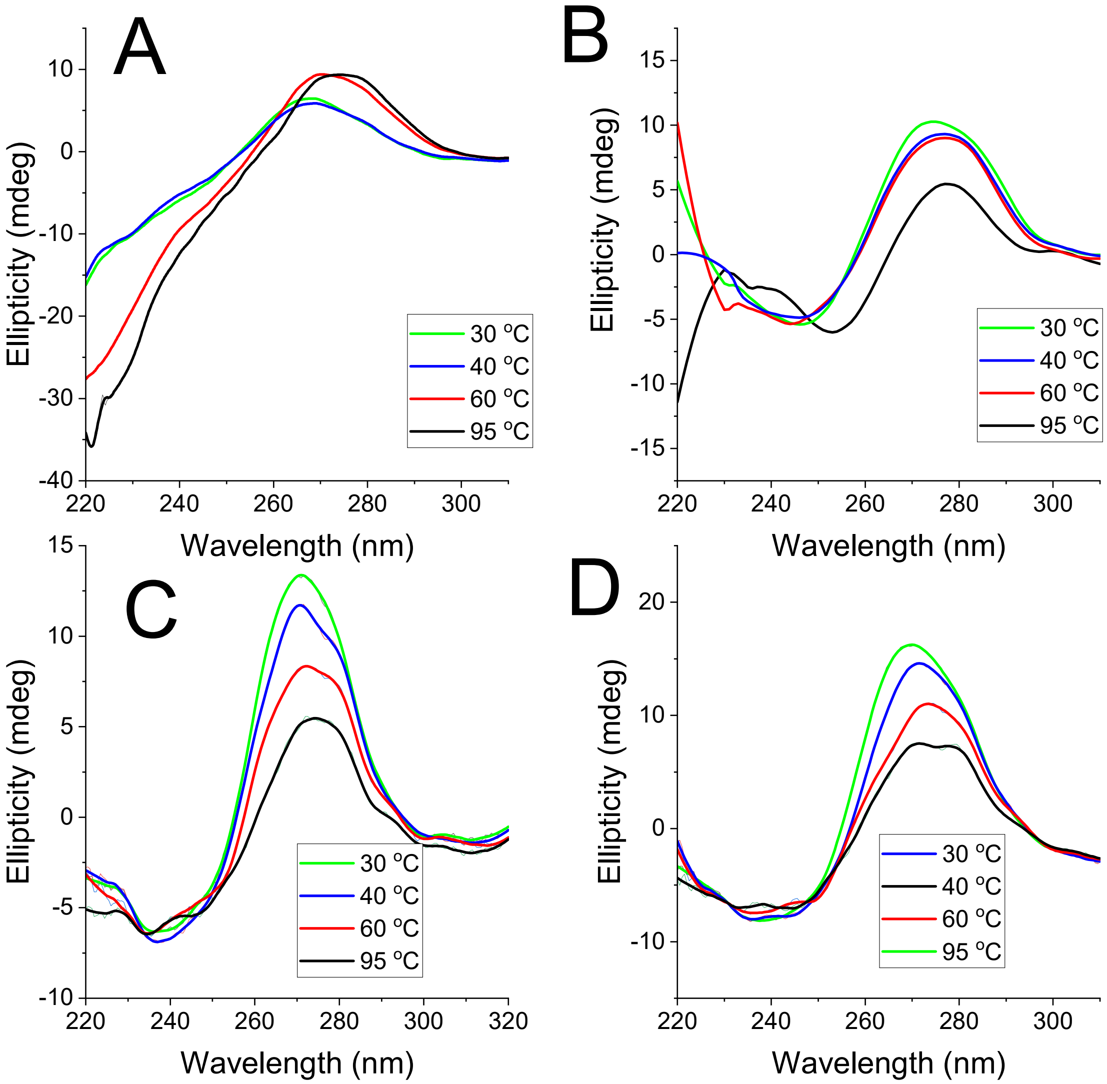

Supplementary Figure 4: Duplicate circular dichroism (CD) spectra of (A) *P. aeruginosa* biofilm, (B) eDNA gel, isolate, and *P. aeruginosa* biofilm cDNA (C) without and (D) with solubilisation in 1-ethyl-3-methylimidazolium and fractional precipitation., at 30 °C (green), 40 °C (blue), 60 °C (red) and 95 °C (black).

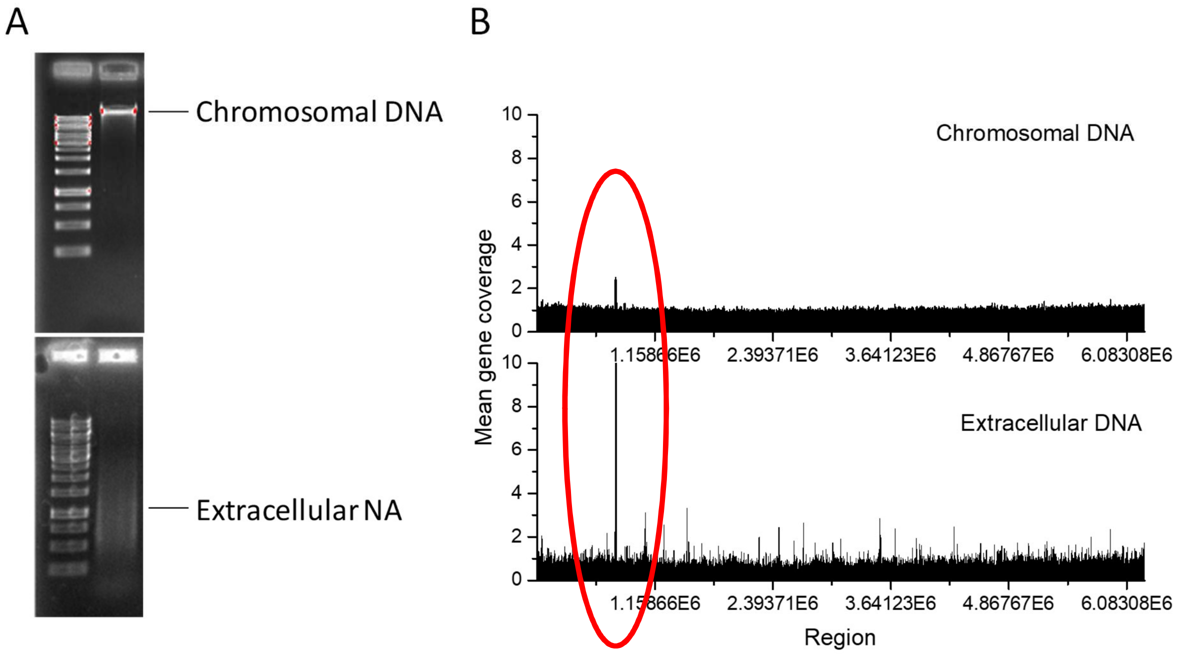

Supplementary Figure 5: Agarose gel loaded with chromosomal DNA extracted from *P. aeruginosa* pre-culture planktonic cells (upper) and extracellular NA gel isolate (lower) (A). Gene coverage of *P. aeruginosa* biofilm chromosomal (upper) and extracellular (lower) DNA normalised against *rpoB* numbers. The red oval denotes the peak resulting from bacteriophage Pf4 genes (B).

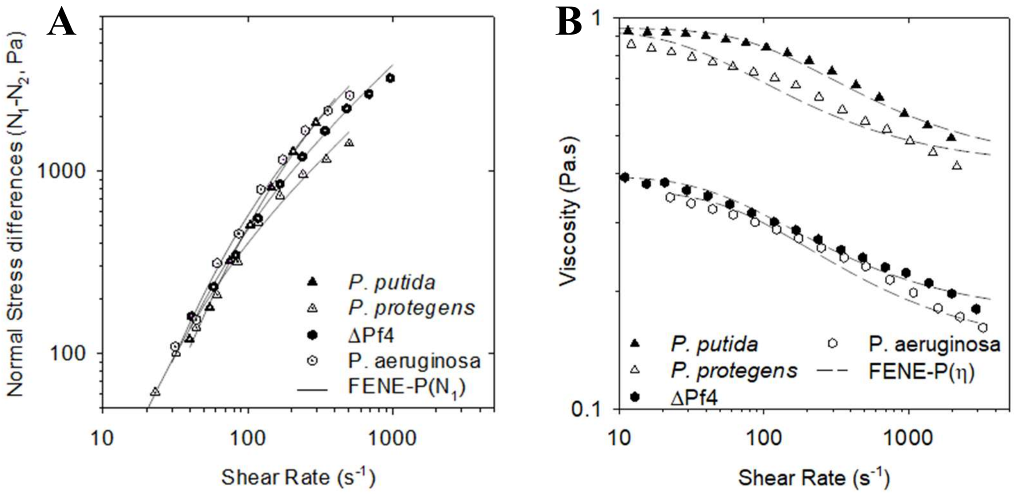

Supplementary Figure 6: (A) Representative normal stress differences (N_1 $-N_2$; n = 2) and (B) viscosities as a function of shear rate for *Pseudomonas* biofilms: *P. aeruginosa, P. putida, P. protogens* and *P. aeruginosa* ΔPf4, dissolved in 1-ethyl-3-methylimidazolium acetate (40 mg/mL) at 25 °C. Lines indicate FENE-P fits to the data.

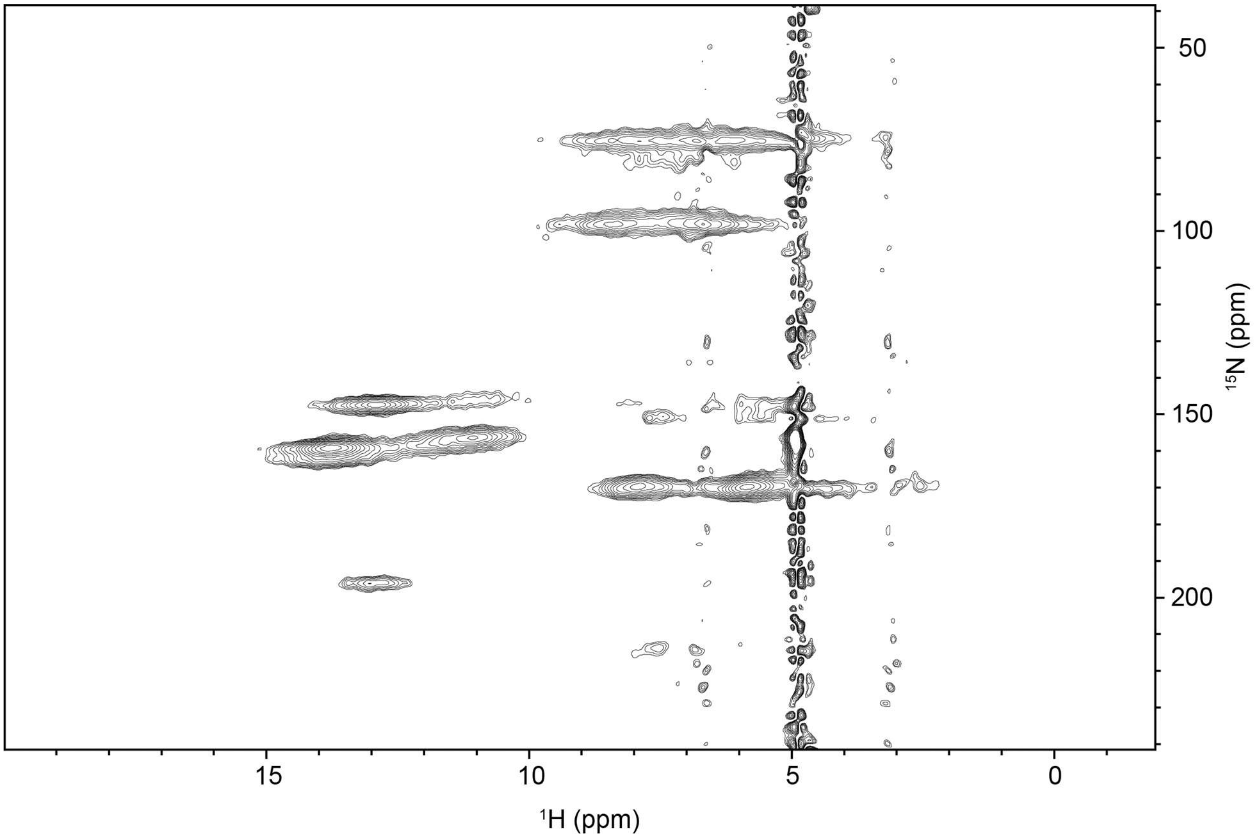

Supplementary Figure 7: Representative (n = 2) solid-state 2D ^{1}H-^{15}N through-space heteronuclear correlation (HETCOR) spectrum of extracellular nucleic acid (NA) gel isolate in double distilled water (2 mg), T = 25 °C.

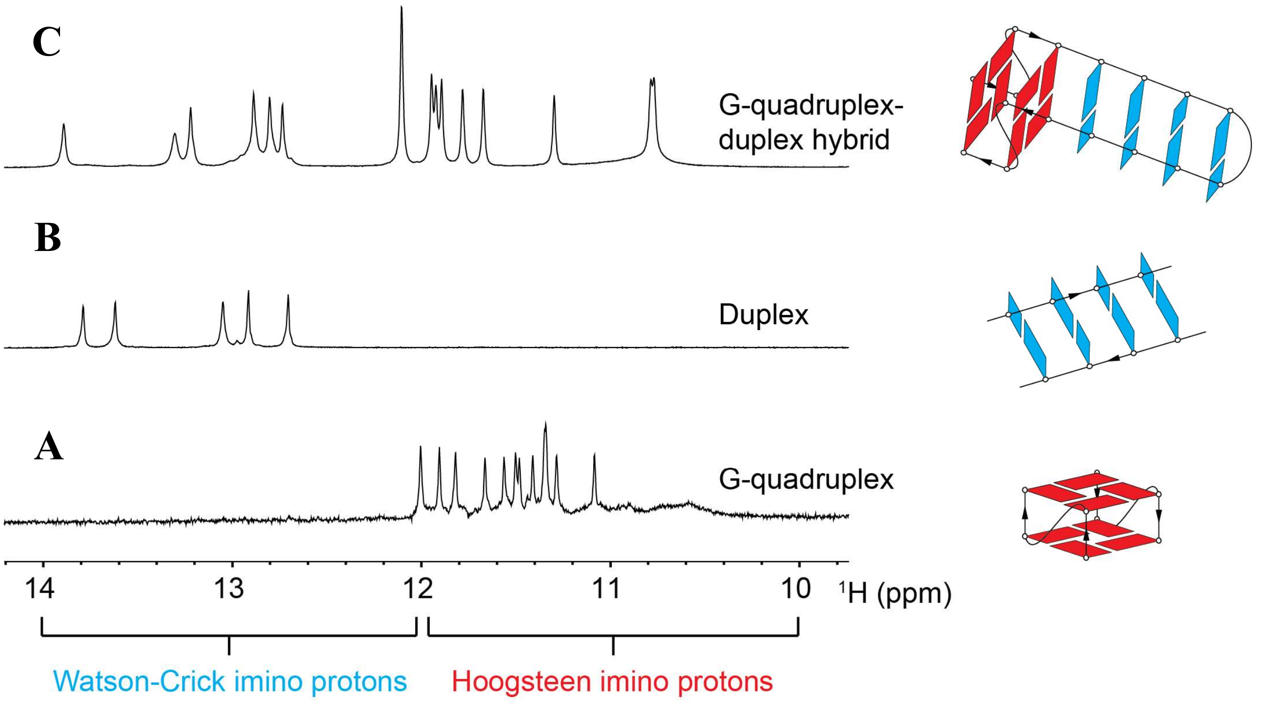

Supplementary Figure 8: 1-D ^{1}H NMR spectra in the imino proton region of well-characterised (A) G-quadruplex (1), (B) duplex (2) and (C) quadruplex-duplex hybrid (3) structures. The 10-12 ppm region shows Hoogsteen-bonded imino protons from non-canonical base-pairs, while the 12-14 ppm region shows Watson-Crick-bonded imino protons from canonical base-pairs. The schematics on the right are for illustration purposes only, demonstrating the structural arrangement in three dimensions.

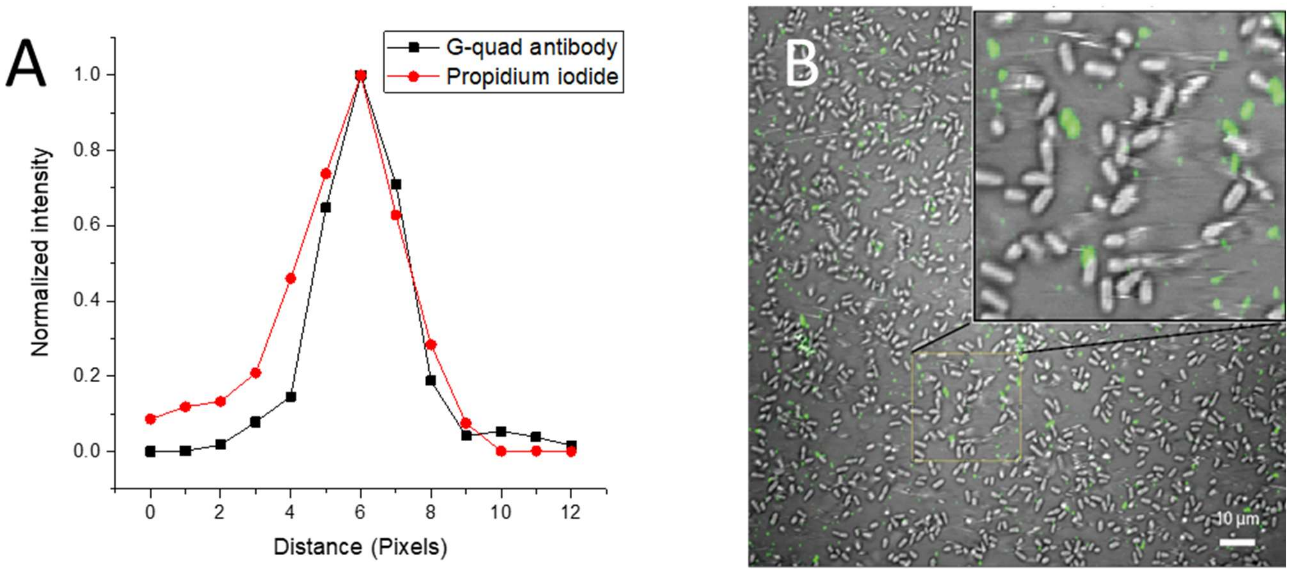

Supplementary Figure 9: (A) Normalised intensity of red (propidium iodide stain) and green (G-quadruplex antibody stain) fluorescence microscopy channels along the horizontal line denoted in the zoomed-in images from Figures 5A-C. (B) Overlapping brightfield and confocal micrograph of *Pseudomonas aeruginosa* rugose small colony variant planktonic cells (18 h, 37 °C, 200 RPM) incubated with anti-DNA G-quadruplex antibody (2 µg/mL) and visualised with GFP-labelled goat anti-mouse IgG, green. The inset region of interest of 6 x 6 µm shows that the G-quadruplex antibody (green) appears outside the cells.

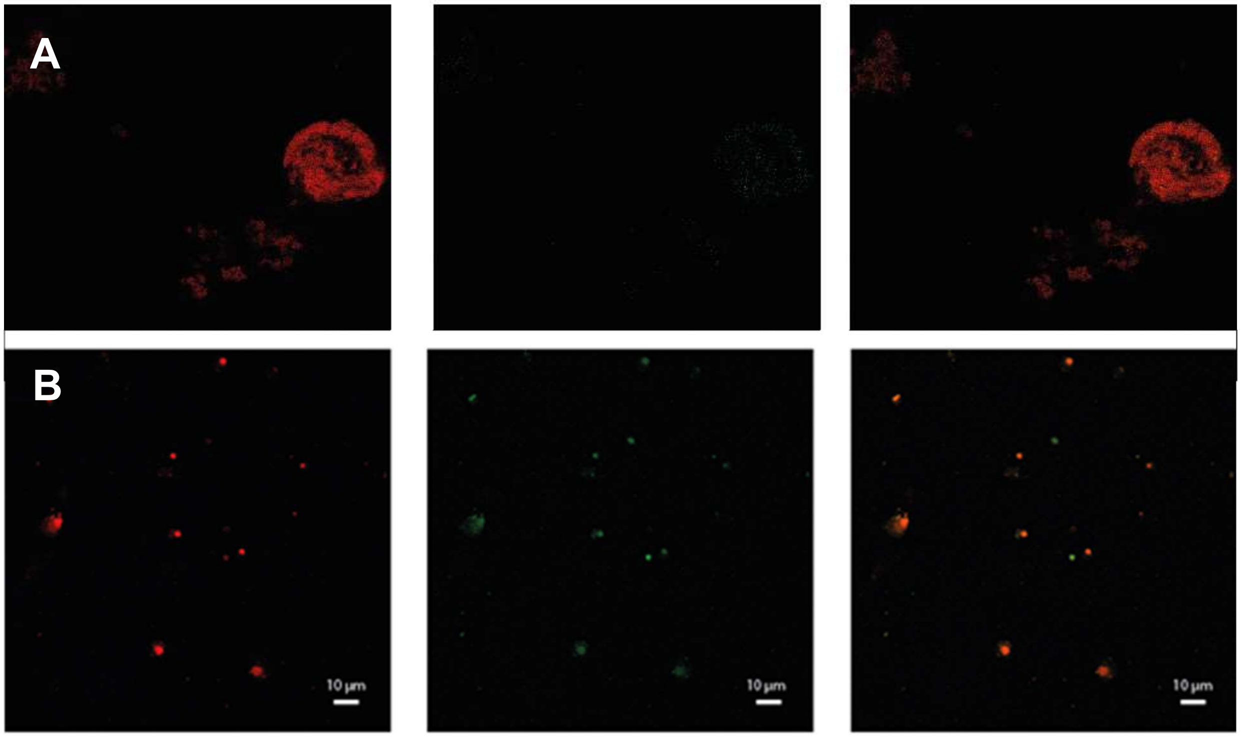

Supplementary Figure 10: Confocal micrographs (Left: propidium iodide, red. Middle: anti-DNA G-quadruplex structures antibody visualised with GFP-labelled goat anti-mouse IgG, green. Right: overlapping) of (A) cDNA of *P. aeruginosa* wild type planktonic cells and (B) well-characterised G-quadruplexing DNA(4) (i.e., positive control), showing that anti-DNA G-quadruplex antibody is specific for G-quadruplex structures.

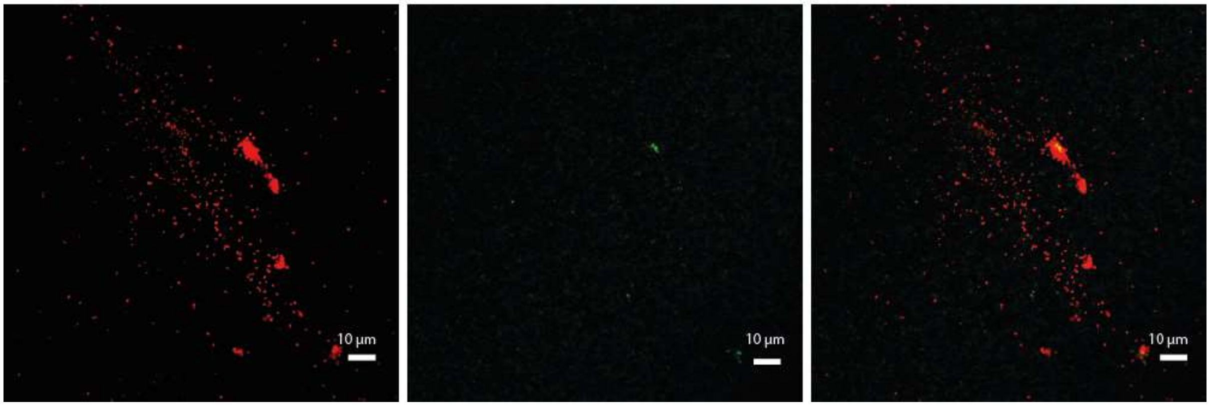

Supplementary Figure 11: Confocal micrographs (Left: propidium iodide (red). Middle: GFP-labelled goat anti-mouse IgG (green). Right: overlapping) of *P. aeruginosa* RSCV pellicle biofilm, showing that anti-mouse IgG does not bind to eDNA fibres in the biofilm matrix.

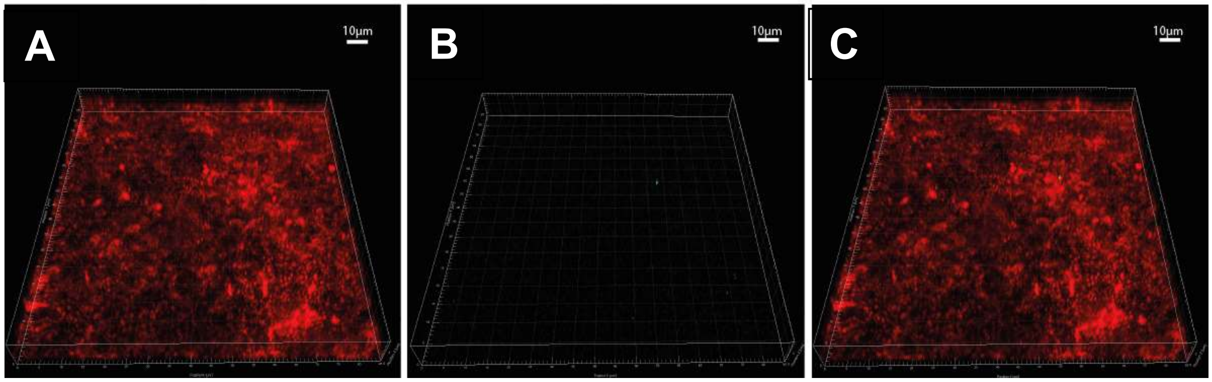

Supplementary Figure 12: Three-dimensional (3-D) confocal micrographs of 3-d *P. aeruginosa* wild type flow cell biofilm showing the binding of DNA-specific dye propidium iodide to the eDNA of the biofilm matrix (PI), (red) (A), the absence of binding of GFP-labelled goat anti-mouse IgG to the eDNA fibres (green) (B) and overlapping binding of both PI and GFP-labelled anti-mouse-IgG (C).

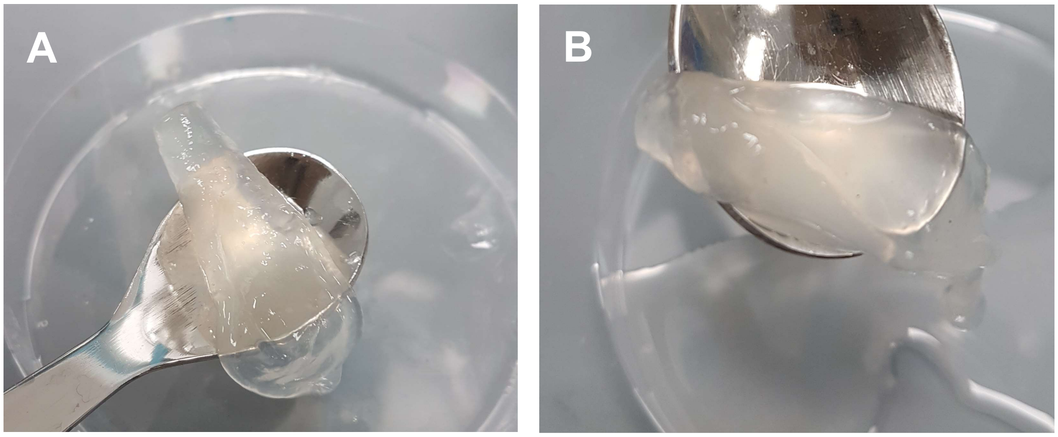

Supplementary Figure 13: Photograph of nucleic acid gel extracted from (A) *P. protegens* and (B) *P. putida*.

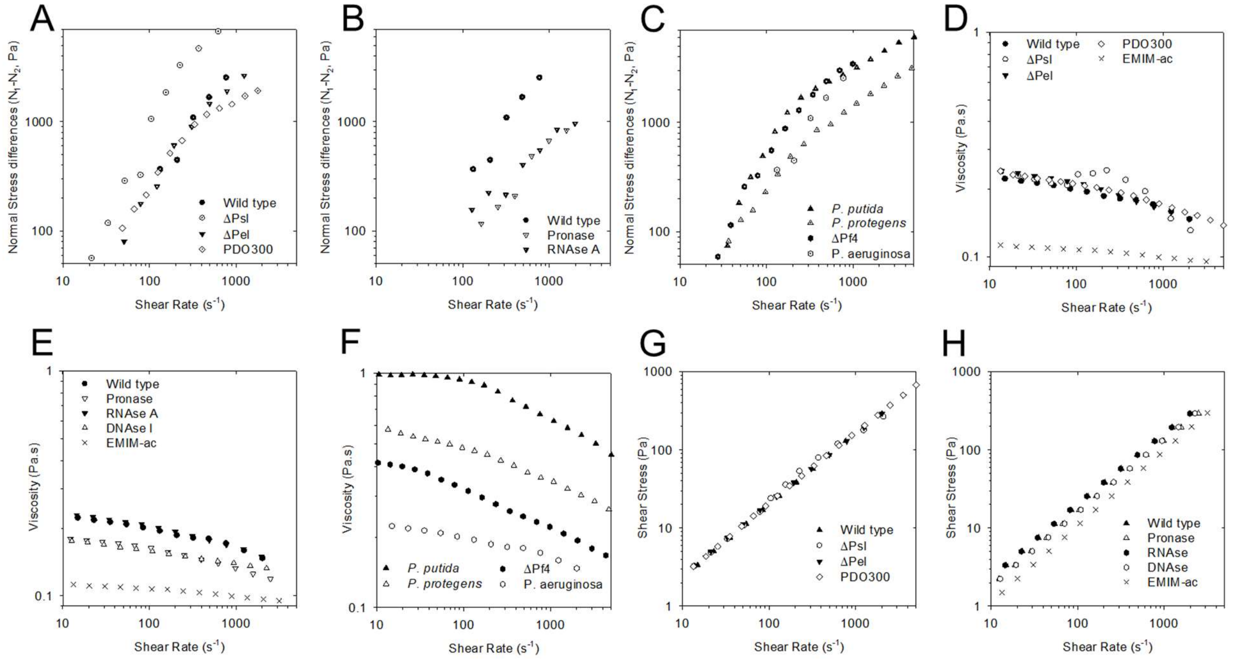

Supplementary Figure 14: Duplicate measurements of (A-C) normal stress difference ($\Delta N = N_1\text{-}N_2$), (D-F) viscosity, and (G-H) shear stress against shear rate. *P. aeruginosa* biofilm wild type, ΔPsl ΔPel and PDO300 (A, D and G), pronase, RNase and DNaseI-digested wild type biofilms (B, E and H), and *P.putida, P. protegens, P. aeruginosa* and *P. aeruginosa* ΔPf4 (C and F) in 1-ethyl-3-methylimidazolium acetate (40 mg/mL) at 25 °C, with 100 µm rheometer measurement gap, and shear stress sweep from 10 to 1000 Pa.

Supplementary Table 1: Power Law exponent (i.e., p in $N_1 = K\dot{\gamma}^p$ and m in $\sigma = K_\sigma \dot{\gamma}^m$) of *P. aeruginosa* biofilms following dissolution in EMIM-Ac. m-values approaching unity indicate a Newtonian-like fluid property. Viscosity is slightly shear thinning (m = 0.8 to 0.9) for all samples except RNase, DNase and EMIM-Ac, which are Newtonian-like ($m \geq 0.93$). This is to be expected from dilute polymer solutions in viscous fluids (i.e., Boger fluids).

	$\sigma = K_\sigma \dot{\gamma}^m$				$N_1 = K_{N1} \dot{\gamma}^n$			
**Sample**	**K_σ**		***m***		**K_{N1}**		***p***	
	**Ave.**	**Std Dev**	**Ave.**	**Std Dev**	**Ave.**	**Std Dev**	**Ave.**	**Std Dev**
P. aeruginosa wild type	0.63	0.08	0.84	0.01	1.44	0.45	1.36	0
ΔPsl	1.26	0.35	0.76	0.04	7.81	5.18	1.21	0.15
ΔPel	0.35	0	0.89	0	0.58	0.15	1.34	0.02
Pronase	0.55	0.03	0.89	0	0.18	0.11	1.64	0.08
RNase	0.34	0.01	0.93	0	2.11	0.09	0.91	0.01
P. putida	1.32	0.01	0.91	0.01	0.56	0.16	1.48	0.08
PDO300	0.73	0.35	0.89	0.01	2.33	0.59	1.10	0.03
P. protegens	1.14	0.01	0.90	0.01	2.75	1.30	1.10	0.11
ΔPf4	0.57	0.02	0.87	0.01	1.59	1.25	1.33	0.24
DNase	0.20	0.01	0.99	0.01	-		-	
EMIM-Ac	0.13	0	0.98	0	-		-	

Supplementary Table 2: Fitting parameters for the FENE-P model including λ_1 = relaxation time, b = a measure of the relative extensibility of the model spring, η_s = solvent viscosity, η_p = polymer contribution to the viscosity. Molecular extensibility and relaxation times, as predicted by FENE-P, decrease in accordance with elasticity.

	**Average**					**Std. Deviation**			
**Sample**	b	λ_1	η_p	η_s		b	λ_1	η_p	η_s
P. aeruginosa wild type	2248.5	0.322	0.260	0.145		643.1	0.066	0.040	0.0026
ΔPsl	3511.5	0.676	0.381	0.161		1000.9	0.219	0.113	0.0114
ΔPel	2000.8	0.227	0.110	0.133		637.8	0.056	0.000	0.0025
Pronase	1871.8	0.103	0.197	0.167		693.1	0.047	0.015	0.0339
RNase	399.2	0.102	0.095	0.183		17.6	0.008	0.003	0.0077
PDO300	909.4	0.453	0.138	0.147		134.2	0.023	0.026	0.0007
DNase	135.7	0.005	0.020	0.129		106.8	0.001	0.011	0.0112
ΔPf4	865.4	0.230	0.243	0.166		129.9	0.034	0.022	0.0072
P. protegens	137.2	0.175	0.501	0.414		19.2	0.028	0.025	0.0013
P. putida	336.9	0.071	0.544	0.440		53.5	0.002	0.018	0.0199

References

1. Do, N.Q. and Phan, A.T. (2012) Monomer–dimer equilibrium for the 5′–5′ stacking of propeller-type parallel-stranded G-quadruplexes: NMR structural study. *Chemistry – A European Journal*, **18**, 14752-14759.
2. Tereshko, V., Minasov, G. and Egli, M. (1999) The Dickerson-Drew B-DNA dodecamer revisited at atomic resolution. *Journal of the American Chemical Society*, **121**, 470-471.
3. Lim, K.W. and Phan, A.T. (2013) Structural basis of DNA quadruplex–duplex junction formation. *Angewandte Chemie International Edition*, **52**, 8566-8569.
4. Phan, A.T., Modi, Y.S. and Patel, D.J. (2004) Propeller-type parallel-stranded G-quadruplexes in the human c-myc promoter. *Journal of the American Chemical Society*, **126**, 8710-8716.
